# Supplementary material for: Single-cell landscape of piglet lung response with Actinobacillus pleuropneumoniae
Source: Virulence. 2026 Mar 17;17(1):2646800. doi: 10.1080/21505594.2026.2646800 (PMC13014559; doi:10.1080/21505594.2026.2646800)
Supplement: zhu et al Manuscript revision Clean.docx [file KVIR_A_2646800_SM1147.docx]

**Single-cell landscape of piglet lung response with *Actinobacillus pleuropneumoniae***

Junhui Zhu^1,2¶^, Sibo Zhu^3¶^, Changyou Xia^4^, Xuan Jiang^1^, Chuntong Bao^1^, Ziheng Li^1^, Rining Zhu^1^, Hexiang Jiang^1^, Fengyang Li^1^, Xiaoguang Zhang^1^, Wei Wang^1^, Hong Chen^1^, Jikun Mei^1^, Jingmin Gu^1^, Na Li^1*^, and Liancheng Lei^1*^

1 State Key Laboratory for Diagnosis and Treatment of Severe Zoonotic Infectious Diseases, Key

Laboratory for Zoonosis Research of the Ministry of Education, Institute of Zoonosis, and College

of Veterinary Medicine, Jilin University, Changchun 130062, China

2 School of Public Health, Beihua University, Jilin 132013, China

3 MOE Key Laboratory of Contemporary Anthropology, School of Life Sciences, Fudan University, Songhu Road 2005, Shanghai 200438, China

4 State Key Laboratory for Animal Disease Control and Prevention, National Poultry Laboratory Animal Resource Center, Heilongjiang Provincial Key Laboratory of Laboratory Animal and Comparative Medicine, Harbin Veterinary Research Institute, Chinese Academy of Agricultural Sciences, Harbin 150069, China

¶These authors contributed equally to this work.

* Corresponding author

E-mails: vetlina2013@126.com; leiliancheng@163.com

**Abstract**

Pulmonary fibrosis is a prevalent, chronic, and fatal illness that poses considerable risks to life and health. *Actinobacillus pleuropneumoniae* (A. pleuropneumoniae) is an archetypal bacteria responsible for inducing significant pulmonary fibrosis, resulting in substantial economic losses in the pig industry. Nevertheless, the immune response in pig lungs against this pathogen and the specific characteristics of fibrosis remain obscure. In this study, single-cell RNA sequencing (scRNA-seq) analysis of piglet lungs with or without A. pleuropneumoniae infection identified 18 subpopulations with different phenotypes.  Monocytes, neutrophils, and plasmacytoid dendritic cells (pDCs) were enriched in the lungs post-infection and responded to infection by boosting IFN-γ-inducible and inflammatory-related gene expression. A. pleuropneumoniae reduces the number of macrophages by inhibiting monocyte differentiation into interstitial macrophages (IM) and alveolar macrophages (AM) and triggering AM endogenous apoptosis. Furthermore, we identified significantly augmented pathological fibroblast-like cells that contributed to the rapid development of pulmonary fibrosis. In contrast, epithelial cells were significantly decreased and included those with features of epithelial-mesenchymal transition differentiated into fibroblasts through the signalling of TGFB1 and HIF1A. Cell-to-cell communication analysis further indicated that the interaction between the epithelial, vascular endothelial, pDCs, and fibroblast subsets, except for COL3A1 fibroblasts, was enhanced mainly via CD74/(COPA or MIF) receptor ligands after infection. Our findings elucidate the key pathogenic mechanisms driving bacterial pneumonia, while establishing a comprehensive molecular resource for developing targeted strategies against *A. pleuropneumoniae* infection and related human fibrotic lung disorders.

**Keyword** Actinobacillus pleuropneumoniae; piglet lung; single-cell RNA sequencing; fibroblast

**Introduction**

Porcine pleuropneumonia is a highly contagious bacterial pneumonia caused by Actinobacillus pleuropneumoniae (A. pleuropneumoniae), which results in massive economic losses to the global pig industry.  A. pleuropneumoniae often causes severe fibrinous hemorrhagic lesions in the lungs, resulting in irreparable and deadly lung pulmonary fibrosis[1]. Furthermore, porcine pleuropneumonia is one of the most complex infections, as other causative pathogens, such as porcine reproductive and respiratory syndrome virus (PRRSV), porcine circovirus type 2 (PCV2), swine influenza virus (SIV), Streptococcus suis, and Glaesserella parasuis are frequently co-infected with A. pleuropneumoniae  [2-4]. Current *A. pleuropneumoniae* vaccines predominantly mitigate clinical manifestations, but exhibit limited efficacy in preventing pathogen colonization and transmission, impeding global eradication efforts for porcine pleuropneumonia [5]. The pathogenesis of *A. pleuropneumoniae* infection progresses through four distinct phases: colonization, nutrient acquisition, evasion of host immune defenses, and tissue necrosis. Key virulence determinants mediating this process include lipopolysaccharides (LPS), capsular polysaccharides, outer membrane proteins (OMPs), and pore-forming Apx toxins [6]. During pulmonary infection, massive neutrophil infiltration occurs in the affected lung tissues, with the subsequent release of neutrophil extracellular traps (NETs) as a bactericidal mechanism. However, *A. pleuropneumoniae* can counteract this defense by secreting nuclease enzymes that specifically degrade NETs, thereby facilitating its immune evasion [7].  *A. pleuropneumoniae* induces macrophage secretion of pro-inflammatory cytokines, including IL-6, IL-1β, TNF-α, IL-8, and MCP-1, which drive hyperinflammatory responses while simultaneously causing macrophage dysfunction, ultimately facilitating bacterial immune evasion [8]. Furthermore, the pathogen interacts with neonatal porcine tracheal epithelial cells, triggering NF-κB pathway activation, IL-8 production, and programmed necrosis of the epithelial barriers [9]. These findings demonstrate that structural and immune cells are critical components of the pulmonary host defense system during *A. pleuropneumoniae* infection. Nevertheless, the molecular interplay between porcine pulmonary tissue cells and *A. pleuropneumoniae* remains poorly characterized at the single-cell level.

Single-cell RNA sequencing (scRNA-seq) has emerged as a transformative tool for delineating tissue ontogeny, tumor microenvironment dynamics, and host-pathogen interplay and is predominantly applied in human and murine systems [10-12]. Its expanding utility in livestock research holds promise for decoding cellular heterogeneity and molecular trajectories underlying complex phenotypes [13-15]. Swine, a promising animal model for immunobiological studies and xenotransplantation research, represents an exclusive natural reservoir for *A. pleuropneumoniae* [16-18]. Single-cell analyses of porcine pulmonary-pathogen interactions may uncover novel immunoregulatory circuits and evolutionarily conserved defence mechanisms. In this study, we used scRNA-seq to systematically analyze the pulmonary immune and structural cell landscape and intercellular communication networks in the lungs of piglets during *A. pleuropneumoniae* infection. Our analysis revealed three hallmark pathological features: (1) multi-mechanistic impairment of lung macrophages, T lymphocyte populations, and epithelial barriers; (2) disproportionate expansion of profibrotic fibroblast subsets exhibiting activated proliferative signatures; and (3) extensive ligand-receptor-mediated interactions between fibroblast subclusters and neighboring cells, particularly involving CD74/COPA signalling axes in epithelial, plasmacytoid dendritic cells (pDCs), and vascular endothelial compartments. Notably, alveolar macrophages (AMs) and alveolar type II (AT II) ATII-2 cells demonstrate relatively quiescent interactions compared to other cellular constituents. These findings provide mechanistic insights into *A. pleuropneumoniae* pathogenesis, while establishing a comprehensive cellular atlas for investigating porcine respiratory disease pathobiology.

**Materials and methods**

**Animal model of A. pleuropneumoniae infection in pig**

Six healthy Rongchang pigs at 45 days of age were purchased from the Harbin Veterinary Research Institute (Chinese Academy of Agricultural Sciences). All animals tested negative for *A. pleuropneumoniae* ApxIV using the ApxIV enzyme-linked immunosorbent (ELISA) kit from Keqian Biology (Keqian Biology, China) and were randomly divided into two groups (A. pleuropneumoniae = 3, Control = 3) (Supplementary additional file 1: Table S1). The A. pleuropneumoniae group was infected by nasal instillation of 1 ml of 1×10^^8^ CFU of A. pleuropneumoniae 5b L20. In contrast, the control group was treated with an equal volume (PBS) volume. The clinical scores of the pigs were assessed using the methodology outlined by Sibila et al. [19], which primarily encompassed evaluations of respiratory distress, appetite loss, and alterations in mental status (Supplementary additional file 1: Table S2). From the day of infection (day 0), the pigs were continuously monitored for clinical signs and body temperature for three consecutive days. At 72 h post-infection, piglets were weighed, euthanized via intravenous injection of sodium pentobarbital (25 mg/kg body weight), and lung tissues were immediately collected from both *A. pleuropneumoniae* infected and PBS-treated control groups. Bacterial loads of bronchoalveolar lavage fluids and lung homogenates were determined by incubation of serial dilutions on brain-heart infusion plates (containing 20 μg/mL NAD (Sigma, USA) and 5% horse serum (Gibco, USA) at 37 °C and colony counting.

**Haematoxylin-and-eosin staining**

Hematoxylin and eosin staining was performed as described [20]. Lung tissues were dehydrated using an ethanol gradient, paraffin-embedded, and sectioned at a thickness of 5 μm. After xylene dewaxing and ethanol hydration, eosin was added for 2 min before washing under running water. Hematoxylin in 1% ethanol and hydrochloric acid was then added for 3 min. After dehydration, the tissues were fixed in an ethanol gradient and xylene. Images were captured using an Olympus microscope (Olympus, Tokyo, Japan).

**Preparation of single-cell suspensions of pig lung tissue**

Lung tissues from three pigs infected with *A. pleuropneumoniae* were promptly excised, focusing on removing 1 cm from the primary area of the lesions caused by *A. pleuropneumoniae*. These lesions were primarily located in the lung parenchyma near the middle of the right diaphragmatic lobe and the distal terminal bronchus. One piece of lung tissue was collected from each pig as an independent biological sample. Lung tissue was selected from healthy pigs, originating from an anatomical location identical to that of the lung tissue collected from pigs infected with *A. pleuropneumoniae*. Subsequently, single-cell suspensions of pig lung tissues were immediately prepared following a previously described standardized experimental protocol [21]. Briefly, the lungs were washed with cold PBS three times and minced into 1 mm pieces using sterilized scissors. Tissue pieces were dissolved in 1640 medium containing 25 U/mL DNase I (Solarbio, China), 300 U/mL collagenase type VIII (Sigma, USA), and 10% fetal bovine serum (FBS) in a 50 mL tube for 20 min at 37 °C. The suspension was mechanically dissociated and filtered through a 70 μm cell strainer. The cell suspensions were pelleted by centrifugation at 350 × g for 7 min at 4 °C. Red blood cell lysis buffer (Solarbio, China) was then applied to remove red blood cells by incubating for 4 min and quenching with 10 ml PBS. The cell suspension was centrifuged at 350 × g for 7 min at 4 °C and the supernatant was decanted. After removing dead cells using OptiPrep medium (Axis-Shield, Scotland), the cells were counted (cell viability=90.67%±2.6583%) and immediately analyzed by scRNA-seq.

**Single-cell RNA library preparation and sequencing**

According to the manufacturer’s protocol, scRNA-seq libraries were prepared using a Chromium single-cell 3ʹ library, gel bead, multiplex kit, and chip kit (10× Genomics). The library pool was sequenced using an Illumina NovaSeq 6000 instrument with 150-base-pair paired-end reads. Subsequently, each sample of the raw sequence data was mapped to the pig reference genome Sscrofa11.1.104 using Cell Ranger v.4.0.0 (10×Genomics). Gene expression matrices were constructed using Cell Ranger Count Function (Genergy, China).

**Sample correlation heatmap and clustering tree**

We generated pseudobulk data by aggregating single-cell raw counts and subsequently computed Pearson correlation coefficients with the top 3,000, 6,000, and 9,000 highly variable genes to assess the relationships between samples. The R package “heatmap” was used to create the sample correlation heatmaps. Additionally, hierarchical cluster analysis was performed by filtering the top 3,000 highly variance genes using the hclust function from the R package “stats”.

**Data quality control and processing of Single-cell RNA-seq**

Raw gene expression matrices were analyzed with the “Seurat” package (v.3.2.2) in R (v.3.6.1) [22]. Briefly, the Seurat object was created using the following criteria: 1) gene expression ≥ 300 genes. 2) Each gene expressed in ≥3 cells. 3) The cells retained-300-4000 genes, unique molecular identifiers (UMIs), and less than 10% of mitochondrial genes. The “DoubletFinder” package removed potential doublets at the expected doublet rate of 0.1. After filtering low-quality cells, gene expression matrices were normalized with a scale factor of 10,000 using the LogNormalize method, and 3,000 genes were selected using the vst method in the FindVariableFeatures function. The linear dimensional reduction algorithm was applied to scaled gene expression matrices using the RunPCA function. Next, we used FindNeighbors and FindClusters (resolution = 0.2) functions to identify the clusters. The RunUMAP procedures were applied for further dimensional reduction. More details about the Seurat analyses can be found on the website tutorial (<https://satijalab.org/seurat/articles/pbmc3k_tutorial.html>).

**Identification and proportional changes of Cell clusters**

The cell clusters were manually annotated based on the differentially expressed genes (DEGs) between clusters using the FindAllMarkers function as previously reported[13, 23-25]. We integrated the cell clusters in each main lineage to perform sub-clustering of T and fibroblast cell populations. ScaleData, RunPCA, RunUMAP, FindClusters, and FindAllMarkers positive marker analyses were run on each main lineage as described above. The identified sub-cluster cell populations were annotated based on their differential gene expression. The changes in the proportions of all cell clusters in each sample from A. pleuropneumoniae infection (n = 3) and control groups (n = 3) were compared. The cell cluster proportions were calculated using prop. table function in R, and a stacked bar chart was generated using ggplot2.

**Immunohistochemistry**

Immunohistochemical staining was performed as previously described with some modifications [26]. Briefly, formalin-fixed, paraffin-embedded lung tissue sections (4 μm) were rehydrated using a graded ethanol series. Heat-mediated antigen retrieval was performed in sodium citrate buffer (10 mM, pH 6.0) at 95 °C for 20 min. Endogenous peroxidase activity was quenched using 3% hydrogen peroxide for 20 min, followed by blocking with 5% donkey serum in PBS for 50 min. Primary antibodies against CD3 (1:200, #MCA5951PB, Bio-Rad) and CD68 (1:200, #25747-1-AP, Proteintech) were applied in an antibody diluent and incubated at 4 °C overnight. Detection was performed using a polymer-based immunohistochemistry kit (MXB Biotechnologies, China) with diaminobenzidine (DAB) chromogen development for 5 min. Sections were counterstained with hematoxylin for 5 min, dehydrated using ascending ethanol concentrations, and cleared in xylene before mounting with neutral resin. Whole-slide digital imaging was performed using a PANNORAMIC MIDI II (3DHISTECH, Budapest, Hungary) automated digital slide scanner. Quantitative analysis of immunoreactivity was performed by measuring the optical density in five randomly selected fields per section using the ImageJ software (v1.53) with the IHC Profiler plugin.

**Flow cytometry**

The cells were washed and resuspended in PBS. The cells were incubated with the zombie NIR fixable viability kit (BioLegend, USA) for 20 min at room temperature. The cells were washed twice with FACS buffer (1% FBS in 1×PBS) and stained with surface antibodies in 100 μL of FACS buffer for 30 min at 4 °C. The following antibodies were used: Pacific blue mouse anti-pig CD45 (1:15) (Bio-Rad, #MCA1222PB, USA), biotin mouse anti-pig CD31 (1:200) (Bio-Rad, #MCA1746B, USA), biotin anti-human CD326 (EPCAM) (1:300) (BioLegend, #324215, USA), FITC mouse anti-human SDC2 (H-7) (1:100) (Santa Cruz, #sc-365624 FITC, USA), FITC mouse IgG1, κ isotype ctrl antibody (1:100) (BioLegend, #400108, USA). The cells were then washed twice with FACS buffer for 5 min 350×g and stained with the secondary antibody Brilliant Violet 605 Streptavidin (1:160) (Biolegend, #405229, USA) for 30 min at 4 °C. Finally, the cells were washed and immediately analyzed using a CytoFLEX Flow Cytometer (Beckman, USA). The data were analyzed with FlowJo v10.6.2 (FlowJo software, BD Biosciences).

**DEG identification and Geneset enrichment analysis**

The DEGs of cells between A. pleuropneumoniae and control groups were calculated using the FindMarkers function of the “Seurat package”. The FindMarkers method uses the Wilcoxon rank-sum test by default. The parameter min. pct was set to 0.25, and the others were set to default. DEGs were selected as avg logFC (Fold-change) ＞0.5 and adjusted *P*-value < 0.05. The “clusterProfiler” R-package was used for Gene Ontology (GO) enrichment analysis [27]. The EnrichGO function used org.Ss.eg.db or org.Hs.eg.db as gene databases, and default parameters were applied.

**Defining cell state scores for feature expression**

Cell scoring measures the expression levels of a specific set of genes in each cell [28]. We applied the R package “AUCell” to calculate pathway activity scores for individual cells. The AUCell Rankings function was used to determine the gene expression ranks for each cell using the default parameters. Each gene list was assigned a score based on the gene set. The AUCell_calcAUC function determines a ranking based on gene expression. The AUC value indicates the percentage of each gene present in the highest-ranked cells, designated as belonging to the pathway genome. Responses to oxidative stress (GO:0006979), inflammatory response (GO:0006954), innate immune response (GO:0045087), and apoptotic signalling pathway (GO:0097190) were used to define the oxidative stress, inflammatory response, and T cell apoptosis score, respectively. All gene sets used in this study are available in additional file 2 supplementary data Table S3.

**Pseudotime trajectory and CytoTRACE analysis**

The pseudotime trajectory analysis for the monocytes and macrophages was run using the R package “Monocle2” [29]. Gene order was selected as variable genes with the criteria of mean_expression>=0.01&dispersion_empirical ≥ 1*dispersion_fit. The branched expression analysis modelling (BEAM) function was used to identify the dynamic gene expression patterns. The branched heatmap was mapped with a q value < 0.0001, and GO enrichment analysis was performed.

We applied the CytoTRACE method to predict the differentiation states of cell clusters in scRNA-seq data [30]. The R package “CytoTRACE” v0.3.3 was used with default parameters to calculate the CytoTRACE scores for the cell clusters. The CytoTRACE scores ranged from 0 to 1, with higher scores indicating a lower degree of differentiation. For further details on the CytoTRACE code, please refer to https://cytotrace.stanford.edu/.

**Sirius red staining**

The Sirius Scarlet Detection Kit (Scientific Phygene, China) was used to stain paraffin-embedded piglet lung sections for 1 h. After staining, sections were cleaned and photographed using an Olympus microscope (Olympus, Japan).

**Cell-cell communication analysis**

CellPhoneDB software was used to infer cell-to-cell ligand-receptor pair interactions as previously reported [31] (<http://www.cellphonedb.org/>). We acquired the QC-filtered raw count matrices and metadata of the cell-type annotation as an input file for the cellphoneDB. In brief, CellphoneDB analysis used the CellphoneDB statistical analysis, plot heatmap_plot, and plot dot_plot commands as the default parameters. The cell-to-cell interaction pairs were selected based on *p*-values < 0.05 to evaluate the relationships between cell types.

**Statistical analysis**

R (version 3.6.1) and GraphPad Prism (version 8.3.0) were used for all statistical analyses and graphs. Differences were considered signiﬁcant at *P* < 0.05.

**Results**

**Overview of piglet lung cell types with and without *A. pleuropneumoniae* infection**

To comprehensively analyze the cell response characteristics of piglet lungs post A. pleuropneumoniae infection. We conducted scRNA-seq on lung cells from pigs infected with 1×10^^8^ CFU of A. pleuropneumoniae for 3 days and healthy pigs for bioinformatics analysis of the obtained data (Figure 1(A)). Clinical scores showed that the control group had no signs of disease, and the A. pleuropneumoniae-infected group showed different clinical scores (1-3 points) (Supplementary Fig S1A, B). On the first day post-infection, sick pigs showed slightly increased body temperature, accelerated breathing, loss of appetite, immobility, lethargy, and diarrhea (Supplementary Fig S1A, B). The CFU of *A. pleuropneumoniae* in the bronchoalveolar lavage fluid and lung homogenate of piglets infected with *A. pleuropneumoniae* were high (Supplementary Fig S1C). The infected lung tissue exhibited noticeable signs of hemorrhagic pneumonia, neutrophil infiltration, epithelial damage, shedding, and significant release of intravascular cellulose (Figure 1(B) and Supplementary Fig S1D). These results indicate that the pigs were infected with A. pleuropneumoniae. Subsequently, we performed scRNA-seq on pig lung tissues of the *A. pleuropneumoniae*-infected and control groups (Supplementary Fig S1E).

ScRNA-seq analysis of uninfected and infected pig lungs revealed 59,062 cells (21,239 in the *A. pleuropneumoniae* group and 37,823 in the control group). To evaluate the similarity between biological replicates within each group (n = 3), pseudo-bulk RNA-seq analysis was performed. Correlation heatmaps and clustering tree analysis revealed robust intra-group transcriptional concordance within both *A. pleuropneumoniae*-infected (n = 3) and control cohorts (n = 3), with distinct inter-group segregation (Supplementary Fig S1F, G). After clustering of uniform approximation and projection (UMAP) analysis, 18 cell types were identified according to their DEGs, i.e., fibroblast-like cells (SDC2^+^CD9^+^COL15A1^+^) (n = 14,937), epithelial (EPCAM^high^S100A2^+^SFTPC^﹣^) (n = 12,316), T cells (CD3E^+^) (n = 9,257), monocyte (CD14^+^FCN1^+^) (n = 4,027), AM (S100A8^+^S100A9^+^) (n = 3,721), proliferating fibroblast (CENPF^+^COL15A1^+^) (n = 2,211), interstitial macrophage (IM) (MARCO^+^CD163^+^) (n = 2,656), B cells (CD79B^+^MS4A1^+^) (n = 2,541), neutrophil (G0S2^+^CXCL8^+^SELL^+^) (n = 1,709), plasma cells (JCHAIN^+^) (n = 1,071), alveolar type II (ATⅡ-1) (EPCAM^low^SFTPC^+^S100A2^﹣^) (n=966), proliferating T cells (TOP2A^+^CD3G^+^) (n = 872), pDCs (PLD4^+^IRF8^+^) (n = 804), lymph endothelial cells (CCL21^+^MMRN1^+^) (n = 824), vascular endothelial cells (VWF^+^PTPRB^+^) (n = 509), COL3A1 fibroblast (COL3A1^+^COL1A2^+^) (n = 451), Unknown (C4BPA^+^HPGD^+^CD3E^+^) (n = 88), and ATⅡ-2 (EPCAM^low^S100A2^+^SFTPC^﹣^) (n = 102) (Figure 1(C-E), Supplementary Fig S2A and Supplementary additional file 3: Table S4).

After *A. pleuropneumoniae* infection, neutrophils and monocytes showed an upward trend, whereas the proportions of AM and IM decreased (Figure 1(F) and Supplementary Fig S2B, C). The percentages of T, B, and plasma cells after infection were relatively reduced (Figure 1(F) and Supplementary Fig S2B, D). Meanwhile, fibroblast-like cells exhibited a significant increase in number post-infection (Figure 1(F) and Supplementary Fig S2B), which was further validated by flow cytometry (Supplementary Fig S2E, F). Conversely, the relative proportion of epithelial cells decreased after the infection (Figure 1(F) and Supplementary Fig S2B). The percentage of ATII subgroups and vascular endothelial cell abundance were relatively low, but the percentage of lymphoid endothelial cells was relatively elevated post-infection (Figure 1(F) and Supplementary Fig S2B). The relative difference in the percentage of immune and structural cells in the piglet lungs with and without *A. pleuropneumoniae* infection suggested that bacterial infection severely dampened the balance of cell populations in the lung tissue.

**Neutrophil, monocyte, and pDCs response *A. pleuropneumoniae* infection by boosting IFN-γ inducible and inflammatory-related gene expression**

Myeloid cells are the major contributor to the innate immunity of piglet lungs after *A. pleuropneumoniae* infection*,* so we further focused on the monocyte, AM, IM, neutrophil, and pDCs cell populations (Figure 1(C) and Figure 2(A)). Neutrophils were activated and had significantly elevated production of the IFN-γ-inducible factors IL-18, IFNGR1, and ISG20 post-infection (Figure 2(B) and Supplementary additional file 4: Table S5). GO analysis revealed significant enrichment of JAK-STAT signalling pathway-associated genes in neutrophils following *A. pleuropneumoniae* infection. In addition, functions such as inflammatory response, cell adhesion, response to lipoprotein stimulation, and positive regulation of leukocyte proliferation were significantly enriched in neutrophils (Figure 2(C) and Supplementary additional file 5: Table S6). Consistent with previous findings that *A. pleuropneumoniae* is resistant to complement-mediated phagocytosis, neutrophils downregulated the expression of bacteria-clearing complement genes C1QA and C1QB (Figure 2(B) and Supplementary additional file 4: Table S5). For monocytes, the expression of genes encoding inflammatory factors IL-18 and NFKB1A, the oxidase SOD2, and the suppressor of cytokine signalling 3 (SOCS3) was upregulated after infection with *A. pleuropneumoniae*(Figure 2(B) and Supplementary additional file 4: Table S5). The expression of pattern recognition receptors TLR2 and TLR4 also tended to increase in monocytes (Supplementary Fig S3). Similar to neutrophils, monocyte GO enrichment profiles were mainly related to the innate immune response and regulation of lymphocyte proliferation (Figure 2(D) and Supplementary additional file 6: Table S7). Upon *A. pleuropneumoniae* infection, pDCs upregulated interferon-inducible lysosomal thiol reductase IFI30, CCR7, and the non-canonical NF-κB subunit RelB (Figure 2(B)). These changes suggest a potential role for pDCs in antimicrobial immune response function. In addition, pDCs were enriched in the positive regulation of RNA metabolic processes, cellular biosynthetic processes, and immune system processes (Figure 2(E) and Supplementary additional file 7: Table S8). The expression of the oxidative stress regulation genes MT1A and SOD2 and the inflammatory gene NFKB1A was upregulated in the AM population post-infection (Figure 2(B)). Following *A. pleuropneumoniae* infection, no significant differences in TLR4 expression were observed in the AM group (Supplementary Fig S3A). IM is a relatively under-studied macrophage that is often classified as an AM-like cell. During *A. pleuropneumoniae* infection, IM upregulated the expression of IFI30 and MT1A while downregulating the IFN-α-induced gene IFI6 (Figure 2(B)). GO enrichment analysis showed that AM and IM cells upregulated similar functions, predominantly those involved in cytoskeleton organization and the oxidative stress response (Figure 2(F, G), Supplementary additional file 8: Table S9 and Supplementary additional file 9: Table S10).

The AUCell scoring system was used to assess innate immune cell inflammation and oxidative stress responses. Neutrophils, monocytes, and pDCs exhibited a highly inflammatory response and a high level of oxidative stress after *A. pleuropneumoniae* infection (Figure 2(H)). In contrast, AM and IM displayed significantly impaired oxidative stress and inflammatory responses, suggesting that *A. pleuropneumoniae* infection may inhibit the immune responses of AM and IM (Figure 2(H)). Thus, these data suggest that neutrophils, monocytes, and pDCs upregulate the oxidative stress response, IFN-γ-inducible factors, and inflammatory factors to boost the immune response to *A. pleuropneumoniae* infection, which was in contrast to AM and IM cell responses.

***A. pleuropneumoniae* reduce the number of macrophages by inhibiting monocyte differentiation into IM and AM and inducing AM endogenous apoptosis**

To understand the mechanism by which *A. pleuropneumoniae* infection is associated with a reduction in lung macrophages, we performed the pseudo-time analysis and found that monocytes could differentiate into AM and IM (Figure 3(A, B)). Subsequently, we clustered the differentially expressed genes along the pseudotime trajectory and identified three clusters (Figure 3(C) and Supplementary additional file 10: Table S11). Cluster 1 comprised monocytes that expressed hallmarks CD14 and FCN1 (Figure 3(C, D)) and was predominantly enriched in myeloid cell differentiation (ZFP36L1), inflammatory response (NFKB1A), wound healing, and smooth muscle cell proliferation (ID2) (Figure 3(C) and Supplementary additional file 11: Table S12). Cluster 2 was IM, and the expression of its marker genes (MARCO and C1QB) gradually increased along the differentiation pathway from monocytes to IM. IM was enriched in complement activation (C1QA, C1QB, and C1QC), myeloid cell differentiation (KLF2 and PRXL2A), and monocyte chemotaxis functions (Figure 3(C, D), Supplementary additional file 10: Table S11 and Supplementary additional file 11: Table S12). Notably, this population was also enriched in the regulation of smooth muscle cell proliferation, and the associated genes AIFI and HMOX1 were highly expressed in IM (Figure 3(C-E) and Supplementary additional file 11: Table S12). Cluster 3 comprised AM, with S100A8 and S100A9 being highly expressed. The functions of the innate immune response, regulation of intrinsic apoptosis signalling pathway, neutrophil chemotaxis, response to the bacterium, and regulation of collagen metabolic process were enriched in this cluster, and the collagen metabolic-related genes CD9 and ACTG1 were highly expressed in AM (Figure 3(C-E) and Supplementary additional file 11: Table S12). In addition, cells in the *A. pleuropneumoniae* infection group were mainly monocytes in the early stages of differentiation. In contrast, the control group was dominated by terminally differentiated IM and AM cells, suggesting that *A. pleuropneumoniae* infection may have inhibited the differentiation of monocytes to IM and AM (Figure 3(F-H)). Notably, pro-apoptotic genes (LGALS1 and LMNA) were upregulated in AM. The anti-apoptotic genes (HMGB2 and BCL2A1) were downregulated at the end of the trajectory (Figure 3(E) and Supplementary additional file 10: Table S11).

***A. pleuropneumoniae* suppresses T cell function by decreasing the number of T lymphocytes and inducing apoptosis of CD8A^+^ γδT cells**

To characterize the role of T cells in A. pleuropneumoniae infection, we identified eight phenotype-specific T cell subsets, that is, putative GNLY^high^CD8^+^T_EM_ (effector memory T cells, T_EM_), CD8A^+^γδT(CD2^+^CD3E^+^CD8A^+^TRDC*^+^*), putative CD8^+^T_RM_ (tissue-resident memory T cells, T_RM_) (CD8^+^T_RM_: XCL1^+^EOMES*^+^*), naïve CD4^+^T (CD4^+^CCR7^+^TCF7^+^SELL^+^), γδT (CD2^-^CD3E^+^CD8A^-^TRDC*^+^*), proliferating T cells (CD3E^+^TOP2A^+^), putative GZMB^hi^ CD8^+^ T_EM_ and CD14^+^CD8^+^ T (CD14^+^ CD8A^+^CD3E^+^) (Figure 4(A-C) and Supplementary additional file 12: Table S13).

Next, we compared the T cell subset percentages and relative numbers in the *A. pleuropneumoniae*-infected and control groups. The fraction of all T cell subsets within the T cell compartment decreased, except for CD8A^+^γδT and proliferating T cells (Figure 4(D)). In healthy pigs, CD8A^﹣^γδ T cells predominated in the resting state, whereas CD8A^+^γδT cells tended to increase relative to  *A. pleuropneumoniae* infection in the percentage of T cells (Figure 4(C, D)). Interestingly, *A. pleuropneumoniae* infection elevated the apoptosis-related genes FOS and LGALS1 in CD8A^+^γδ T cells and downregulated the ribosomal protein genes RPL38*,* RPS21*,* RPS13, and RPS17 (Figure 4(E) and Supplementary additional file 13: Table S14). The expression of the immune checkpoint molecule LAG3 was significantly upregulated following infection, suggesting potential T-cell exhaustion (Figure 4(F)) [32]. Moreover, the apoptosis signalling pathway score of CD8A^+^γδT cells significantly increased post-infection, indicating that *A. pleuropneumoniae* induced apoptosis of CD8A^+^ γδT cells (Figure 4(G)). GO analysis further showed that CD8A^+^γδT was involved in the regulation of actin cytoskeleton organization and supramolecular fiber organization, indicating the potential of CD8A^+^γδT in fibrosis pathogenesis (Figure 4(H) and Supplementary additional file 14: Table S15). Additionally, post-*A. pleuropneumoniae* infection, GNLY^high^CD8^+^T_EM_, CD8A^+^γδT, CD8^+^T_RM_, γδT, and proliferating T cells significantly elevated the AUCell innate immune response score, suggesting their potential capacity to regulate the early immune response (Figure 4(G)). GO functional analysis of the upregulated DEGs of T subsets after *A. pleuropneumoniae* infection was also enriched in pathways related to the immune response process, T cell activation, response to bacteria, and granulocyte chemotaxis. (Figure 4(H) and Supplementary additional file 14: Table S15).

***A. pleuropneumoniae* infection increases TGFB1 and HIF1A expression and promotes epithelial differentiation into fibroblasts**

Epithelial cells were the most abundant structural cells in the lung, and *A. pleuropneumoniae* infection significantly damaged the alveolar structure and lung epithelial cells (Figure 1(B)). Here, we identified three epithelial cell subsets: SFTPC^+^S100A2^-^ATⅡ-1, SFTPC^+^S100A2^+^ ATⅡ-2, epithelial cells, and EPCAM^high^S100A2^+^SFTPC^-^ (Figure 5(A)). Epithelial cells undergo epithelial-to-mesenchymal transition and play an important role in fibrotic diseases [33, 34]. Epithelial cells and ATⅡ-2 showed similar phenotypes, expressing S100A2, S100A8, S100A9, CD9, and SDC2 (Figure 1(E)). However, there were few AGER and AQP5 labeled alveolar type I (ATⅠ) cells, most likely due to the damage to ATⅠ cells caused by *A. pleuropneumoniae* infection and the low number of ATⅠ cells in physiological states [13, 35]. Compared with the control group, the relative percentages of all epithelial subsets decreased after *A. pleuropneumoniae* infection, among which epithelial cells were the most significant (Figure 1(F)). Epithelial-mesenchymal transition is a key step in pulmonary fibrosis. After *A. pleuropneumoniae* infection, the expression of epithelial cell markers such as EPCAM and FABP5 was reduced in epithelial cells. In contrast, the expression of fibroblast-specific marker genes such as SDC2 and COL15A1 was enhanced (Figure 5(B)). UMAP analysis further indicated that epithelial cells were connected to the fibroblasts. Monocle and CytoTRACE analysis indicated that epithelial cells could differentiate into fibroblast-like cells, suggesting that epithelial cells are an essential source of fibroblasts (Figure 5(A, B), and Supplementary Fig S4A-C). Furthermore, previous studies identified a COL1A1 expressing epithelial subset exclusively in human pulmonary fibrosis [36]. The pathological extracellular matrix genes COL1A1, COL2A1, and COL3A1 were highly expressed in epithelial cells post-*A. pleuropneumoniae*infection (Figure 5(C)).

In addition, we found that *A. pleuropneumoniae* infection tended to increase the expression of TGFB1, HIF1A, COPS5, and OS9 in epithelial cells, promoting epithelial cell transformation (Figure 5(D, E)). DGE analysis of epithelial cells revealed that MT1A, MT3, GP91-PHOX, S100A13, and CXCR4 were up-regulated after infection (Figure 5(F) and Supplementary additional file 15: Table S16). GO analysis indicated that epithelial cells were primarily enriched in the function of ATP metabolism, amide biosynthesis, and cellular respiration. ATII cell subsets were mainly enriched in cellular metabolism and mRNA processing (Figure 5(G) and Supplementary additional file 16: Table S17).

**Heterogeneous function of the fibroblast in piglet lung post *A. pleuropneumoniae* infection**

Fibroblasts heavily infiltrated the piglet lung following A. pleuropneumoniae infection, among which fibroblast-like cells were the most prominent (Figure 1(F) and Supplementary Fig S2B, D-E), suggesting that A. pleuropneumoniae infection could induce pulmonary fibrosis. To evaluate the degree of pulmonary fibrosis, we performed Sirius red staining and confirmed that the collagen fibers after A. pleuropneumoniae infection tended to increase compared to the control group (Figure 6(A)). To further focus on fibroblast heterogeneity and its role in fibrosis during A. pleuropneumoniae infection, we pooled all fibroblasts to identify five fibroblast subgroups: MT1A fibroblasts (MT1A^+^MT3^+^), HBEGF fibroblasts (HBEGF^+^), IFN fibroblasts (CXCL10^+^CXCL9^+^IL18^+^), proliferating fibroblasts (CENPF^+^TOP2A^+^), and COL3A1 fibroblasts (COL3A1^+^COL1A2^+^COL1A1^+^) (Figure 6(B-D), Supplementary additional file 17: Table S18 and Supplementary Fig S5A). After A. pleuropneumoniae infection, MT1A fibroblasts and HBEGF fibroblasts were the predominant fibroblast subgroups; however, the relative fraction of IFN fibroblasts declined, and other cell populations were not altered (Figure 6(E)).

Next, we explored the transcriptional characteristics of MT1A fibroblasts. DGE analysis revealed that the expression of metallothionein-related genes, MT1A and MT3, was upregulated after *A. pleuropneumoniae* infection (Figure 6(F) and Supplementary additional file 18: Table S19). In addition, GO analysis indicated that MT1A fibroblasts were enriched in metal ion regulatory activities and oxidative responses following infection (Figure 6(G), Supplementary Fig S5B, Supplementary additional file 19: Table S20 and Supplementary additional file 20: Table S21). The antigen presentation-related genes HLA-DRA, SLA-DQB1, and CD74 were upregulated in *A. pleuropneumoniae*-infected HBEGF fibroblasts, suggesting that HBEGF fibroblasts have antigen presentation functions and are involved in the innate immune response (Figure 6(H)). In addition, HBEGF fibroblasts showed increased expression of the insulin-inducible gene INSIG1, which can promote fibrosis (Figure 6(F) and Supplementary additional file 18: Table S19). Further enrichment analyses of HBEGF fibroblasts showed upregulation of genes whose functions are associated with the inflammatory immune pathway (NF-κB), innate immune response, LPS stimulation response, and smooth muscle cell proliferation (Figure 6(G) and Supplementary additional file 19: Table S20). IFN fibroblasts can regulate the inflammatory response, innate immune response, and external stimuli, and enhance the expression of MT1A and SDC2 fibroblast marker genes after *A. pleuropneumoniae* infection. IFN fibroblast cells showed decreased expression of CXCL9 and CXCL10, reducing their ability to recruit cytotoxic lymphocytes (CTLs), natural killer (NK) cells, and macrophages [37] (Figure 6(F)). Interestingly, proliferating fibroblasts enhanced the expression of fibrosis genes SDC2, COL15A1, and INSIG1 decreased the expression of EPCAM during infection (Figure 6(F, I)). Moreover, UMAP analysis showed that proliferating fibroblasts were associated with fibroblast-like and epithelial cells, and pseudo-temporal trajectory analysis indicated that proliferating fibroblasts differentiated into fibroblast-like cells, indicating that fibroblast-like cells may be derived from these proliferating fibroblasts and epithelia (Figure 1(C) and Supplementary Fig S5C). Finally, COL3A1 fibroblasts are a subset of lung fibroblasts that are more prevalent in lung infections. COL1A1 and COL3A1 fibroblasts were identified as pathogenic extracellular matrices. After *A. pleuropneumoniae*infection, the matrix remodeling genes COL1A1, COL1A2, COL3A1, FN1, DCN, LUM, and VCAN were all increased (Supplementary Fig S5D). GO functional analysis indicated that this population was enriched regarding lung epithelial growth, TGFB stimulation, and collagen fibrous organization (Figure 6(G)). GO analysis of the human dataset indicated that HBEGF fibroblasts, IFN fibroblasts, and COL3A1 fibroblasts promoted smooth muscle cell proliferation and tissue repair (Supplementary Fig S5B). In addition, TLR2 expression was elevated in HBEGF fibroblasts, indicating that fibroblasts might sense *A. pleuropneumoniae* infection (Supplementary Fig S5E).

**Epithelial, vascular endothelial, and pDCs interact with fibroblast subset through the CD74/COPA receptor-ligand pair, except for COL3A1 fibroblast**

Cell-cell communication networks were constructed among all the cell populations in the porcine lung using CellPhoneDB. Under physiological conditions, epithelial cells strongly interacted with ATII-2 cells (Figure 7(A)). However, *A. pleuropneumoniae* infection diminished the contact between epithelial cells and ATII-2 while enhancing the interaction with all fibroblast subsets, suggesting that epithelial cells were undergoing a robust process of fibroblast transformation (Figure 7(A,B)). Consistent with the findings that numerous cell subsets promoted the proliferation and collagen formation of myofibroblasts, cell interaction analysis indicated that epithelial, vascular endothelia, lymph endothelial, ATII-1, pDCs, monocytes, T cells, B cells, IM, neutrophils, plasma, and proliferating T cells promoted the interaction with fibroblast subsets (Figure 7(A,B)), among these, epithelial, vascular endothelial, and pDCs had the most apparent interactions (Figure 7(B)). These findings suggest that *A. pleuropneumoniae*-triggered fibrinopurulent pleuropneumonia injury may arise primarily from interactions between pulmonary cells and fibroblasts during infection. Intriguingly, AM did not boost the association with each subgroup of fibroblasts following *A. pleuropneumoniae* infection, but instead marginally decreased intercellular connections (Figure 7(A, B)).

Next, we investigated the interactive receptor pairs between each subpopulation of fibroblasts, epithelial cells, vascular endothelial cells, and pDCs. The interactions between epithelial cells and MT1A fibroblasts, HBEGF fibroblasts, IFN fibroblasts, and proliferating fibroblasts were mediated primarily by CD74/COPA, followed by CD74/MIF and EGFR/MIF receptors (Figure 7(C)). Epithelial cells strengthened communication with T, lymph endothelial, and ATII-1 cells via the CD74/COPA ligand pair after *A. pleuropneumoniae* infection, whereas COL3A1 fibroblasts and neutrophils were unaffected (Figure 7(D)). Notably, COL3A1 fibroblasts differed from other fibroblast subpopulations, which primarily enhanced contact with epithelial cells through EGFR/TGFB1, EGFR/MIF, and ICAM1/ITGAL, further indicating that *A. pleuropneumoniae*-induced fibroblasts were highly heterogeneous and exerted distinct fibrosis development (Figure 7(C)). Further analysis showed that in addition to COL3A1 fibroblasts, other fibroblast subsets and vascular endothelial cells predominantly communicated through CD74/COPA, CXCL8/ACKR1, and TGFB1/TGF beta receptor1 (Figure 7(E)). Intriguingly, fibroblast subsets increase contact with ACKR1^+^ vascular endothelial cells through CXCL8/ACKR1 to engage in the process of leukocyte recruitment and promote mesenchymal transition in thyroid-associated eye illness [38]. These results indicate that vascular endothelial cells are involved in the inflammatory response and fibrosis after*A. pleuropneumoniae* infection. Comparative analyses revealed conserved molecular pathways involved in porcine and human fibrotic progression. pDCs preferentially mediated intercellular crosstalk with fibroblast subpopulations (excluding COL3A1-enriched subsets) through the CD74/MIF, CD74/COPA, and TGFB1/TGFbeta receptor1 signalling axes during infection. In contrast, COL3A1 fibroblasts exhibited EGFR-centric interactions with pDCs, predominantly via the EGFR/MIF and EGFR/TGFB1 ligand receptors (Figure 7(F)).

**Discussion**

The immune response mounted by immune cells against infection represents a core mechanism for controlling various fatal complications and tissue damage. Previous studies have identified that immune cells，including antigen-presenting neutrophils, CD4^+^ T cells, and CD163^+^ monocytes counter *A. pleuropneumoniae* infection through diverse regulatory pathways[39-41]. In this study, we observed that following *A. pleuropneumoniae* infection, neutrophils, monocytes, and pDCs upregulated the release of IFN-γ-inducible factors (e.g., IL-18 and ISGs) along with other inflammatory mediators. These findings provide clearer insight into the cellular source of IL-18 during anti-*A. pleuropneumoniae* infection than earlier reports[42]. In recent years, research has also revealed that structural cells (such as fibroblasts, epithelial cells, and endothelial cells) can perform immune-related functions including pathogen recognition and antigen presentation in coordination with classical immune cells[43]. Using scRNA-seq, we identified an excessively proliferating fibroblast-like cell population. Further subcluster analysis revealed multiple previously uncharacterized fibroblast subpopulations linked to *A. pleuropneumoniae* pathogenicity and host defense, which exhibited extensive cell-cell communication networks with various immune cells. Nevertheless, the precise functional mechanisms of these cellular populations warrant further investigation. Collectively, this work systematically delineates the immune response landscape of both immune and structural cells following *A. pleuropneumoniae* infection, thereby laying a groundwork for future research into porcine *A. pleuropneumoniae* pathogenesis.

The host innate immune defense against acute pulmonary bacterial infection requires effective responses that involve the recruitment and activation of neutrophils and monocytes that migrate to the lungs [44, 45]. Analysis of scRNA-Seq data revealed that during *A. pleuropneumoniae* infection, monocytes, pDCs, and neutrophils were rapidly recruited to the lung and exerted antimicrobial functions, partly through upregulation of interferon-stimulated genes (ISGs) and IL-18. The latter is the only cytokine substantially raised in the peripheral CD163^+^ monocytes of *A. pleuropneumoniae*-infected pigs, which enhances neutrophil activation [41]. The ability of neutrophils to kill *A. pleuropneumoniae* is stronger than that of alveolar macrophages (AM) because the bacterium upregulates the expression of copper-zinc superoxide dismutase (Cu/Zn SODs) in AM, which helps it escape AM killing [46]. Our data also showed significant upregulation of superoxide dismutase SOD2 in AM, which may weaken AM’s bactericidal ability. In addition, macrophages undergo endogenous apoptosis, resulting in fewer macrophages post infection. Interestingly, IM promoted the proliferation of smooth muscle cells, and AM upregulated the process of collagen metabolism, indicating that macrophages may be involved in the process of pulmonary fibrosis. We also observed that fibroblast-like cells acquired macrophage-specific surface markers (CD163, S100A8, and S100A9). This phenotypic convergence implies that *A. pleuropneumoniae* infection may drive the macrophage-to-fibroblast transition, which is consistent with previous studies [47, 48]. Furthermore, the pro-inflammatory cytokine IL-18 synergistically enhanced IFN-γ production, amplifying JAK-STAT-mediated immune activation. This cascade demonstrates that *A. pleuropneumoniae* triggers a robust host defense response that is predominantly orchestrated by monocytes, neutrophils, and pDCs.

Unlike humans and mice, the fraction of γδ T cells is high in porcine tissues, facilitating fast T cell responses to external stimuli [49]. We also found that CD8A^+^γδ T cells were highly enriched in the porcine lungs. However, our scRNA-seq analysis revealed significant T cell depletion following *A. pleuropneumoniae* infection. This phenomenon was consistent with observations in other respiratory infections (e.g., *K. pneumoniae*, influenza virus, SARS-CoV-2) and could be attributed to severe inflammatory cytokine stimulation[50]. Further analysis revealed that following *A. pleuropneumoniae* infection, CD8A^+^γδ T cells showed marked activation and an upregulation of apoptotic genes. This pro-apoptotic shift provides a plausible mechanism for the subsequent depletion of T cells. Recent studies indicate that Th17 cell abundance correlates with chronic lung damage following *A. pleuropneumoniae* infection[51], suggesting the key roles of T cell subsets in the development of the disease. Consistent with these findings, our data further suggest that T cells may play a potential regulatory role in orchestrating the early immune response against *A. pleuropneumoniae*. Interestingly, CD8^+^T_RM_ and CD8^+^T_EM_ cell populations may not be specific to *A. pleuropneumoniae*-induced memory, but may represent T_RM_ and T_EM_ residents in lung tissue. Studies have suggested that resident T_RM_ and T_EM_ in lung tissue to combat pathogen infection can initiate non-specific antibacterial responses, coordinate immune reactions, and recruit T, B, and NK cells to trigger pathogen alarms [52]. However, the specific roles of porcine T_RM_ and T_EM_ require further investigation. Intriguingly, T cells showed enhanced regulatory activities associated with structural cytoskeleton repair and collagen metabolism, indicating that T cells might play a role in improving *A. pleuropneumoniae*-induced pulmonary fibrosis. Previous studies have shown that T cells display aberrant damage and create severe lung fibrotic lesions after COVID-19 infection, consistent with our findings [11, 53].

Fibroblasts are critical mesenchymal cells in the submucosa that are not expected to interact with microorganisms in the airway. However, once the alveolar macrophage and epithelial barrier is compromised, bacteria may invade fibroblasts in the lung interstitium. This invasion may trigger a compensatory host response, wherein fibroblasts upregulate Toll-like receptors as part of the innate immune sensing [43, 54]. We found that HBEGF fibroblasts highly expressed TLR2, as did proliferating fibroblasts post *A. pleuropneumoniae* infection, suggesting an antimicrobial inflammatory response in fibroblasts. HBEGF fibroblasts increased the expression of antigen-presenting genes such as HLA-DRA, SLA-DQB1, and CD74. This suggests that HBEGF fibroblast, as well as IFN fibroblasts, exhibited distinct immune barrier function in the context of *A. pleuropneumoniae* infection. Additionally, the genes associated with proliferation of smooth muscle cells and collagen formation were upregulated in MT1A fibroblasts, HBEGF fibroblasts, IFN fibroblasts, and COL3A1 fibroblasts, suggesting that these subsets may promote the progression of pulmonary fibrosis.

Research has established that *A. pleuropneumoniae* infection induces severe pulmonary fibrosis, a hallmark pathology that is a major cause of mortality[55]. Our results showed that fibroblasts constituted the predominant cellular compartment (70.89%) in infected lungs, likely driven by TGFB1 and HIF1A mediated epithelial-mesenchymal transition and fibroblast proliferation. In addition, IFN fibroblasts also significantly regulate the proliferation of smooth muscle cells, indicating that these IFN fibroblasts may also contribute to this pathological development. Moreover, the cell populations with fibrotic characteristics in *A. pleuropneumoniae* infection were similar to those in influenza virus and SARS-CoV-2 infection [26, 56]. We discovered that interaction characteristics induced by *A*. *pleuropneumoniae* infection were highly analogous to those found in idiopathic fibrosis. Specifically, most of pulmonary cell types enhanced interactions with fibroblast subsets following *A*. *pleuropneumoniae* infection. Notably, epithelial cells, vascular endothelial cells, and pDCs exhibited the strongest crosstalk with fibroblasts. Furthermore, CD74/COPA and MIF are the most critical interaction ligand pairs between these cells and fibroblasts, indicating that they may be effective targets for inhibiting fibrotic progression. To prove these interactions, further spatial transcriptomics is required. Based on the similarities between fibrinous pneumonia caused by *A. pleuropneumoniae* and the symptoms of human pulmonary fibrosis, pigs appear to be perfect models[57]. However, many of the sub-clusters identified in porcine data require more in-depth studies.

**Conclusion**

We established a single-cell resolution atlas of piglet lungs with and without *A. pleuropneumoniae* infection, revealing that macrophage dysfunction, epithelial cell injury, T cell apoptosis, and fibroblast hyperproliferation collectively drive infection-induced pathological damage. This comprehensive cellular atlas defines porcine pulmonary cellular composition and function with unprecedented precision, and provides mechanistic insights into bacterial pneumonia pathogenesis. Notably, the observed fibrotic mechanisms establish pigs as translational models for human pulmonary fibrosis research.

**Acknowledgments**

We thank Professor Paul R Langford from the Section of Pediatric Infectious Disease, Imperial College London, for helping us with language editing. We also thank Dr. Faming Zhao (Huazhong University of Science and Technology) for generously sharing his experience in statistical analysis.

**Disclosure statement**

The authors declare that they have no known competing ﬁnancial interests or personal relationships that could have inﬂuenced the work reported in this study.

**Author contributions**

Liancheng Lei: Conceptualization, Funding acquisition, writing–review, and editing. Na Li: Conceptualization, Funding acquisition, writing–review and editing, project administration. Junhui Zhu: Methodology, Visualization, Investigation, Writing-Original Draft, Writing-Review and Editing. Sibo Zhu: Methodology, Visualization, Data Curation. Changyou Xia: Resources, Methodology, Investigation, Formal analysis. Xuan Jiang: Methodology, Formal analysis, and investigation. Chuntong Bao: Methodology, Investigation. Ziheng Li: Methodology, Data Curation. Rining Zhu: Methodology. Hexiang Jiang: Methodology, Visualization. Fengyang Li: Supervision, Writing-Review and Editing. Xiaoguang Zhang: Investigation. Wei Wang: Methodology, Investigation. Hong Chen: Investigation. Jikun Mei: Data Curation. Jingmin Gu: Formal analysis.

**Funding**

This study was supported by National Natural Science Foundation of China (Grant No. 32573370; Grant No. 32102686; Grant No.31520103917).

**Data availability statement**

The scRNA-seq data generated in this study were deposited in the NCBI Gene Expression Omnibus database (GEO) under accession code GSE231494 <https://www.ncbi.nlm.nih.gov/geo/query/acc.cgi?acc=GSE231494> (Two groups: APP = 3, Control = 3). The code for single-cell data analysis in this research is available through this URL: <https://github.com/DC-Jun/Single-cell-landscape-of-piglet-lung-response-with-Actinobacillus-pleuropneumoniae> (DOI:10.5281/zenodo.15856403). The supplementary materials and raw data associated with this study can be accessed on <https://doi.org/10.6084/m9.figshare.29633870>[58].

**Ethics statement**

All animal experiments were performed in strict accordance with the guidelines and regulations of the Animal Research and Ethics Committees of the Harbin Veterinary Research Institute, Chinese Academy of Agricultural Sciences (200811-3). Animal License Number (SYXK 2022-005). All animal studies were conducted in accordance with the ARRIVE guidelines (ARRIVE checklist: <https://doi.org/10.6084/m9.figshare.29633870>).

**ORCID**

Liancheng Lei <https://orcid.org/0000-0002-5872-1842>

**References**

[1] Negrete-Abascal E, Reyes M E, Garcia R M, et al. Flagella and Motility in Actinobacillus pleuropneumoniae [J]. J Bacteriol, 2003; 185(2): 664-8. doi:10.1128/JB.185.2.664-668.2003

[2] Qi W, Zhu R, Bao C, et al. Porcine circovirus type 2 promotes Actinobacillus pleuropneumoniae survival during coinfection of porcine alveolar macrophages by inhibiting ROS production [J]. Vet Microbiol, 2019; 233: 93-101. doi:10.1016/j.vetmic.2019.04.028

[3] Czyzewska-Dors E, Dors A, Kwit K, et al. Pig Lung Immune Cytokine Response to the Swine Influenza Virus and the Actinobacillus Pleuropneumoniae Infection [J]. J Vet Res, 2017; 61(3): 259-65. doi:10.1515/jvetres-2017-0036

[4] Saade G, Deblanc C, Bougon J, et al. Coinfections and their molecular consequences in the porcine respiratory tract [J]. Vet Res, 2020; 51(1): 80. doi:10.1186/s13567-020-00807-8

[5] Ramjeet M, Deslandes V, Gouré J, et al. Actinobacillus pleuropneumoniae vaccines: from bacterins to new insights into vaccination strategies [J]. Anim Health Res Rev, 2008; 9(1): 25-45. doi:10.1017/s1466252307001338

[6] Brogaard L, Klitgaard K, Heegaard P M, et al. Concurrent host-pathogen gene expression in the lungs of pigs challenged with Actinobacillus pleuropneumoniae [J]. BMC Genomics, 2015; 16(1): 417. doi:10.1186/s12864-015-1557-6

[7] de Buhr N, Bonilla M C, Pfeiffer J, et al. Degraded neutrophil extracellular traps promote the growth of Actinobacillus pleuropneumoniae [J]. Cell Death Dis, 2019; 10(9): 657. doi:10.1038/s41419-019-1895-4

[8] Ramjeet M, Deslandes V, St Michael F, et al. Truncation of the lipopolysaccharide outer core affects susceptibility to antimicrobial peptides and virulence of Actinobacillus pleuropneumoniae serotype 1 [J]. J Biol Chem, 2005; 280(47): 39104-14. doi:10.1074/jbc.M502852200

[9] Auger E, Deslandes V, Ramjeet M, et al. Host-pathogen interactions of Actinobacillus pleuropneumoniae with porcine lung and tracheal epithelial cells [J]. Infect Immun, 2009; 77(4): 1426-41. doi:10.1128/IAI.00297-08

[10] Ren X, Wen W, Fan X, et al. COVID-19 immune features revealed by a large-scale single-cell transcriptome atlas [J]. Cell, 2021; 184(7): 1895-913 e19. doi:10.1016/j.cell.2021.01.053

[11] Melms J C, Biermann J, Huang H, et al. A molecular single-cell lung atlas of lethal COVID-19 [J]. Nature, 2021; 595(7865): 114-9. doi:10.1038/s41586-021-03569-1

[12] Kalucka J, de Rooij L, Goveia J, et al. Single-Cell Transcriptome Atlas of Murine Endothelial Cells [J]. Cell, 2020; 180(4): 764-79 e20. doi:10.1016/j.cell.2020.01.015

[13] Wang F, Ding P, Liang X, et al. Endothelial cell heterogeneity and microglia regulons revealed by a pig cell landscape at single-cell level [J]. Nat Commun, 2022; 13(1): 3620. doi:10.1038/s41467-022-31388-z

[14] Zhang L, Zhu J, Wang H, et al. A high-resolution cell atlas of the domestic pig lung and an online platform for exploring lung single-cell data [J]. J Genet Genomics, 2021; 48(5): 411-25. doi:10.1016/j.jgg.2021.03.012

[15] Alfieri J M, Wang G, Jonika M M, et al. A Primer for Single-Cell Sequencing in Non-Model Organisms [J]. Genes (Basel), 2022; 13(2). doi:10.3390/genes13020380

[16] Fairbairn L, Kapetanovic R, Sester D P, et al. The mononuclear phagocyte system of the pig as a model for understanding human innate immunity and disease [J]. J Leukoc Biol, 2011; 89(6): 855-71. doi:10.1189/jlb.1110607

[17] Madhusoodanan J. After the First Pig-to-Human Heart Transplant, Scientists Look to the Future of Cardiac Xenotransplantation [J]. JAMA, 2022; 328(20): 1999-2001. doi:10.1001/jama.2022.15434

[18] Griffith B P, Goerlich C E, Singh A K, et al. Genetically Modified Porcine-to-Human Cardiac Xenotransplantation [J]. N Engl J Med, 2022; 387(1): 35-44. doi:10.1056/NEJMoa2201422

[19] Sibila M, Aragón V, Fraile L, et al. Comparison of four lung scoring systems for the assessment of the pathological outcomes derived from Actinobacillus pleuropneumoniae experimental infections [J]. BMC Vet Res, 2014; 10: 165. doi:10.1186/1746-6148-10-165

[20] Wang S, Zheng Y, Li J, et al. Single-Cell Transcriptomic Atlas of Primate Ovarian Aging [J]. Cell, 2020; 180(3): 585-600 e19. doi:10.1016/j.cell.2020.01.009

[21] Slyper M, Porter C B M, Ashenberg O, et al. A single-cell and single-nucleus RNA-Seq toolbox for fresh and frozen human tumors [J]. Nat Med, 2020; 26(5): 792-802. doi:10.1038/s41591-020-0844-1

[22] Stuart T, Butler A, Hoffman P, et al. Comprehensive Integration of Single-Cell Data [J]. Cell, 2019; 177(7): 1888-902 e21. doi:10.1016/j.cell.2019.05.031

[23] Raredon M S B, Adams T S, Suhail Y, et al. Single-cell connectomic analysis of adult mammalian lungs [J]. Sci Adv, 2019; 5(12): eaaw3851. doi:10.1126/sciadv.aaw3851

[24] Herrera-Uribe J, Wiarda J E, Sivasankaran S K, et al. Reference Transcriptomes of Porcine Peripheral Immune Cells Created Through Bulk and Single-Cell RNA Sequencing [J]. Front Genet, 2021; 12: 689406. doi:10.3389/fgene.2021.689406

[25] Zhu Z, Mao R, Liu B, et al. Single-cell profiling of African swine fever virus disease in the pig spleen reveals viral and host dynamics [J]. Proc Natl Acad Sci U S A, 2024; 121(10): e2312150121. doi:10.1073/pnas.2312150121

[26] Wang S, Yao X, Ma S, et al. A single-cell transcriptomic landscape of the lungs of patients with COVID-19 [J]. Nat Cell Biol, 2021; 23(12): 1314-28. doi:10.1038/s41556-021-00796-6

[27] Yu G, Wang L G, Han Y, et al. clusterProfiler: an R package for comparing biological themes among gene clusters [J]. OMICS, 2012; 16(5): 284-7. doi:10.1089/omi.2011.0118

[28] Corridoni D, Antanaviciute A, Gupta T, et al. Single-cell atlas of colonic CD8(+) T cells in ulcerative colitis [J]. Nat Med, 2020; 26(9): 1480-90. doi:10.1038/s41591-020-1003-4

[29] Qiu X, Mao Q, Tang Y, et al. Reversed graph embedding resolves complex single-cell trajectories [J]. Nat Methods, 2017; 14(10): 979-82. doi:10.1038/nmeth.4402

[30] Gulati G S, Sikandar S S, Wesche D J, et al. Single-cell transcriptional diversity is a hallmark of developmental potential [J]. Science, 2020; 367(6476): 405-11. doi:10.1126/science.aax0249

[31] Efremova M, Vento-Tormo M, Teichmann S A, et al. CellPhoneDB: inferring cell-cell communication from combined expression of multi-subunit ligand-receptor complexes [J]. Nat Protoc, 2020; 15(4): 1484-506. doi:10.1038/s41596-020-0292-x

[32] Bretz A C, Parnitzke U, Kronthaler K, et al. Domatinostat favors the immunotherapy response by modulating the tumor immune microenvironment (TIME) [J]. J Immunother Cancer, 2019; 7(1): 294. doi:10.1186/s40425-019-0745-3

[33] Zhang J, Tian X J, Zhang H, et al. TGF-β-induced epithelial-to-mesenchymal transition proceeds through stepwise activation of multiple feedback loops [J]. Sci Signal, 2014; 7(345): ra91. doi:10.1126/scisignal.2005304

[34] Xu Y, Mizuno T, Sridharan A, et al. Single-cell RNA sequencing identifies diverse roles of epithelial cells in idiopathic pulmonary fibrosis [J]. JCI Insight, 2016; 1(20): e90558. doi:10.1172/jci.insight.90558

[35] Parimon T, Yao C, Stripp B R, et al. Alveolar Epithelial Type II Cells as Drivers of Lung Fibrosis in Idiopathic Pulmonary Fibrosis [J]. Int J Mol Sci, 2020; 21(7). doi:10.3390/ijms21072269

[36] Habermann A C, Gutierrez A J, Bui L T, et al. Single-cell RNA sequencing reveals profibrotic roles of distinct epithelial and mesenchymal lineages in pulmonary fibrosis [J]. Sci Adv, 2020; 6(28): eaba1972. doi:10.1126/sciadv.aba1972

[37] Tokunaga R, Zhang W, Naseem M, et al. CXCL9, CXCL10, CXCL11/CXCR3 axis for immune activation - A target for novel cancer therapy [J]. Cancer Treat Rev, 2018; 63: 40-7. doi:10.1016/j.ctrv.2017.11.007

[38] Li Z, Wang M, Tan J, et al. Single-cell RNA sequencing depicts the local cell landscape in thyroid-associated ophthalmopathy [J]. Cell Rep Med, 2022; 3(8): 100699. doi:10.1016/j.xcrm.2022.100699

[39] Li N, Zhu J, Chen P, et al. High-dimensional analysis reveals an immune atlas and novel neutrophil clusters in the lungs of model animals with Actinobacillus pleuropneumoniae-induced pneumonia [J]. Vet Res, 2023; 54(1): 76. doi:10.1186/s13567-023-01207-4

[40] Bao C, Jiang X, Tian Y, et al. IL-21-dependent Ly6C(+)Ly6G(+)CD4(+) T cells found in lung enhance macrophages function against Actinobacillus pleuropneumoniae infection in mice [J]. Cell Death Discov, 2025; 11(1): 440. doi:10.1038/s41420-025-02742-z

[41] Jarosova R, Ondrackova P, Leva L, et al. Cytokine expression by CD163+ monocytes in healthy and Actinobacillus pleuropneumoniae-infected pigs [J]. Res Vet Sci, 2022; 152: 1-9. doi:10.1016/j.rvsc.2022.07.015

[42] Bao C, Liu B, Zhu R, et al. IFN-gamma(-/-) Mice Resist Actinobacillus pleuropneumoniae Infection by Promoting Early Lung IL-18 Release and PMN-I Accumulation [J]. Infection and Immunity, 2021; 89(6). doi:10.1128/iai.00069-21

[43] Krausgruber T, Fortelny N, Fife-Gernedl V, et al. Structural cells are key regulators of organ-specific immune responses [J]. Nature, 2020; 583(7815): 296-302. doi:10.1038/s41586-020-2424-4

[44] Ondrackova P, Nechvatalova K, Kucerova Z, et al. Porcine mononuclear phagocyte subpopulations in the lung, blood and bone marrow: dynamics during inflammation induced by Actinobacillus pleuropneumoniae [J]. Vet Res, 2010; 41(5): 64. doi:10.1051/vetres/2010035

[45] Ondrackova P, Leva L, Kucerova Z, et al. Distribution of porcine monocytes in different lymphoid tissues and the lungs during experimental Actinobacillus pleuropneumoniae infection and the role of chemokines [J]. Vet Res, 2013; 44(1): 98. doi:10.1186/1297-9716-44-98

[46] Langford P R, Loynds B M, Kroll J S. Cloning and molecular characterization of Cu,Zn superoxide dismutase from Actinobacillus pleuropneumoniae [J]. Infect Immun, 1996; 64(12): 5035-41. doi:10.1128/iai.64.12.5035-5041.1996

[47] Yang F, Chang Y, Zhang C, et al. UUO induces lung fibrosis with macrophage-myofibroblast transition in rats [J]. Int Immunopharmacol, 2021; 93: 107396. doi:10.1016/j.intimp.2021.107396

[48] Zhuang T, Chen M H, Wu R X, et al. ALKBH5-mediated m6A modification of IL-11 drives macrophage-to-myofibroblast transition and pathological cardiac fibrosis in mice [J]. Nat Commun, 2024; 15(1): 1995. doi:10.1038/s41467-024-46357-x

[49] Gerner W, Mair K H, Schmidt S. Local and Systemic T Cell Immunity in Fighting Pig Viral and Bacterial Infections [J]. Annu Rev Anim Biosci, 2022; 10: 349-72. doi:10.1146/annurev-animal-013120-044226

[50] Zhang S, Zhang N, Han J, et al. Dynamic immune status analysis of peripheral blood mononuclear cells in patients with Klebsiella pneumoniae bloodstream infection sepsis using single-cell RNA sequencing [J]. Front Immunol, 2024; 15: 1380211. doi:10.3389/fimmu.2024.1380211

[51] Sassu E L, Ladinig A, Talker S C, et al. Frequency of Th17 cells correlates with the presence of lung lesions in pigs chronically infected with Actinobacillus pleuropneumoniae [J]. Vet Res, 2017; 48(1): 4. doi:10.1186/s13567-017-0411-z

[52] Kalia V, Sarkar S, Ahmed R. CD8 T-cell memory differentiation during acute and chronic viral infections [J]. Adv Exp Med Biol, 2010; 684: 79-95. doi:10.1007/978-1-4419-6451-9_7

[53] Gu W, Gan H, Ma Y, et al. The molecular mechanism of SARS-CoV-2 evading host antiviral innate immunity [J]. Virol J, 2022; 19(1): 49. doi:10.1186/s12985-022-01783-5

[54] Hutton A J, Warner J A, Staples K J. Manning the Barricades: Lung Fibroblasts and CD4+ T Cells as the Last Line of Defense against Bacterial Invasion? [J]. Crit Rev Immunol, 2018; 38(5): 367-78. doi:10.1615/CritRevImmunol.2018026611

[55] Merialdi G, Dottori M, Bonilauri P, et al. Survey of pleuritis and pulmonary lesions in pigs at abattoir with a focus on the extent of the condition and herd risk factors [J]. Vet J, 2012; 193(1): 234-9. doi:10.1016/j.tvjl.2011.11.009

[56] Boyd D F, Allen E K, Randolph A G, et al. Exuberant fibroblast activity compromises lung function via ADAMTS4 [J]. Nature, 2020; 587(7834): 466-71. doi:10.1038/s41586-020-2877-5

[57] Vergara-Alert J, Rodon J, Carrillo J, et al. Pigs are not susceptible to SARS-CoV-2 infection but are a model for viral immunogenicity studies [J]. Transbound Emerg Dis, 2021; 68(4): 1721-5. doi:10.1111/tbed.13861

[58] Zhu J, N Li, Lei L, et al. Single-cell landscape of piglet lung response with Actinobacillus pleuropneumoniae. July 2025. doi:10.6084/m9.figshare.29633870.v1

**Figure Legends**

**Figure 1. Single-cell landscape of the piglet lung with or without** ***A. pleuropneumoniae* infection.** (A) The flowchart describes the general experimental design of this study. ScRNA-seq was used to obtain a transcriptional profile of the piglet lung. This ﬁgure was created with BioRender.com. (B) Haematoxylin-and-eosin-stained lung sections from the pigs with or without *A. pleuropneumoniae* infection. (C) A UMAP plot shows the different cell clusters. (D) A UMAP plot shows the origins of cell clusters. (E) A dot plot displays the indicated marker expression of cell clusters in panel C. (F) Average proportion of each cell clusters derived from control (n = 3) and *A. pleuropneumoniae* (n = 3). Bar plot shows relative cell compositions at a single sample level.

**Figure 2. Immune response of myeloid cells with *A. pleuropneumoniae* infection.** (A) Myeloid cell (monocyte, AM, IM, neutrophil, and pDCs) clusters highlighting in UMAP projection. (B) The differential gene expression analysis of myeloid cells of *A. pleuropneumoniae* in comparison with the control. An adjusted p-value < 0.05 is labeled in red, while an adjusted p-value ≥0.05 is labeled in black. (C-G) GO enrichment analyses for the upregulated genes in *A. pleuropneumoniae* compared with the control. Panel C: neutrophil, panel D: monocyte, panel E: pDCs, panel F: AM and panel G: IM. (H) Violin plots showing AUCell score levels of inflammatory response and oxidative stress in the indicated cell clusters between *A. pleuropneumoniae* and control groups. Two-sided Wilcox test.

**Figure 3.** **Trajectory analysis of monocytes, IM and AM in *A. pleuropneumoniae* and control samples.** (A-B) Cells in the tree are colored by the cluster assignment (A) and pseudotime (B). (C) Heatmap showing the functional gene sets and GO terms in monocytes, AM and IM. (D-E) Proﬁling of dynamic expression of CD14, FCN1, MARCO, C1QB, S100A8, and S100A9 (D) and AIF1, HMOX1, CD9, ACTG1, LGALS1, LMNA, BCL2A1, and HMGB2 (E) along the trajectories in AM (solid line) and IM (dotted line) clusters. (F) Density plots showing the dynamic number of monocytes, AM and IM along the trajectory. (G) Density plots showing the number of monocytes, AM and IM along the trajectory stratiﬁed for *A. pleuropneumoniae* vs control, respectively. (H) Density plots showing the dynamic number of monocyte-to-macrophage along the trajectory stratiﬁed for *A. pleuropneumoniae* vs control.

**Figure 4.** **Immunological features of T cell subsets.** (A) UMAP plot showing the distribution of T cell subsets. (B) UMAP plot showing the origins of T cells. (C) Stacked violin plots showing expression of canonical cell markers. (D) Average proportion of T cell subclusters derived from control (n = 3) and *A. pleuropneumoniae* (n = 3). Bar plot shows relative T cell subclusters compositions at a single sample level. (E) Scatter-plot showing differential gene expression in the CD8A^+^ γδ T. (F) Violin plot showing the expression of exhausted gene LAG3 in CD8A^+^ γδ T cell. (G) Violin plots showing AUCell score levels of apoptotic signaling pathway and innate immune response in T subsets derived from *A. pleuropneumoniae* and control groups. two-sided wilcox test. (H) Bubble plot displaying the representative GO enrichment terms of the up-regulated genes in *A. pleuropneumoniae* compared with control in T cell subsets. two-sided Wilcox test.

**Figure 5. Characterization of lung injury and epithelial–mesenchymal transition.** (A) A UMAP plot highlighting ATⅡ-1, ATⅡ-2, epithelia and fibroblast-like clusters. (B) Violin plots of EPCAM, FABP5, SDC2 and COL15A1 mRNA expression in Epithelia cells from *A. pleuropneumoniae* and control groups. (C) Bubble plot showing the expression COL1A1, COL1A2 and COL3A1 in Epithelia cells from *A. pleuropneumoniae* and control samples. (D-E) Violin plots of TGFB1(D), HIF1A, OS9 and COPS5 (E) mRNA expression in epithelia cells from *A. pleuropneumoniae* and control groups. (F) Scatterplot showing different gene expression in the epithelia cells. (G) Bubble plot revealing the representative GO enrichment terms of the up-regulated genes from *A. pleuropneumoniae* compared with control in ATⅡ-1, ATⅡ-2 and epithelia cells.

**Figure 6. Characterization of piglet lung fibroblasts post *A. pleuropneumoniae* infection.** (A) Sirius red staining of lung tissues in control and *A. pleuropneumoniae* groups. (B) A UMAP plot showing the distribution of fibroblast cell subsets. (C) A UMAP plot displaying the origins of fibroblast cell subsets. (D) A dot plot displays the indicated marker expression of cell clusters in panel B.(E) Average proportion of each subset derived from control and *A. pleuropneumoniae* groups. (F) The differential gene expression analysis of fibroblast subsets of *A. pleuropneumoniae* in comparison with control. An adjusted p-value < 0.05 is labeled in red, while an adjusted p-value ≥0.05 is labeled in black. (G) A bubble plot showing the representative GO enrichment terms of the upregulated genes from *A. pleuropneumoniae* compared with control in fibroblast subsets. (H) Violin plots of HLA-DRA, SLA-DQB1 and CD74 mRNA expression in HBEGF fibroblast from *A. pleuropneumoniae* and control. (I) Violin plots of SDC2, COL15A1, and EPCAM mRNA expression in proliferating fibroblast from *A. pleuropneumoniae* and control.

**Figure 7. Cell-communication of fibroblast subsets with other subsets in pleuropneumonia.** (A-B) Heatmap showing the interaction intensity among major cell subsets in the lung from control group (A) and *A. pleuropneumoniae* group (B). (C, E-F) Bubble chart showing the interaction of ligand pairs between epithelial cells (C), vascular endothelial cells (E) and pDCs (F) and all fibroblast subsets in individuals in the control and *A. pleuropneumoniae* group, respectively. On the Y-axis, each row represents the name of a receptor ligand pair. The X-axis depicts the interaction between fibroblast cells subsets and epithelial cells, pDCs, and vascular endothelial cells, respectively (The intercellular communication is bidirectional, indicating that one cell (e.g., cell A) can have an impact on another cell (e.g., cell B), then conversely, cell B can also affect on cell A). The color of the bubbles shows the average expression level of the two genes in the interacting cell groups; the redder the color, the higher the expression level. Meanwhile, the size of the bubble corresponds to the -log10 value of the P value, indicating the significance of the interaction; the larger the bubble, the more significant. (D) The interaction of epithelial cells with major cell subsets from individuals with control and *A. pleuropneumoniae* by CD74/COPA.

**Supplementary figures**

**Figure S1. Pathological changes and** **data quality control.** (A) A line graph shows body temperature changes between control and *A. pleuropneumoniae* infected group. (B) Trends in clinical signs scores of *A. pleuropneumoniae*-infected pigs. (C) Bacterial counts in BALF and lung homogenate in *A. pleuropneumoniae* infected lung. (D) Pathological changes of the lungs in *A. pleuropneumoniae*-infected pigs. This ﬁgure was created with BioRender.com. (E) Barcode Rank Plots of 6 single cell libraries were generated from *A. pleuropneumoniae*-infected lung tissue and control healthy pig lung samples. (F) Sample similarity heatmap generated from pseudobulk gene expression data. Darker color indicates higher similarity between samples. (G) Hierarchical tree presenting the relationship between samples using the top 3000 highly variable genes.

**Figure S2. Characteristics of cell clusters proportion changes.** (A) UMAP plots of canonical markers for all cell types in the lung. (B) A UMAP plot displaying the origins of all cell clusters from individuals with control and *A. pleuropneumoniae*. (C, D) Immunohistochemistry staining of the macrophage cell marker CD68 and T cell marker CD3E in the lungs of the Control and *A. pleuropneumoniae* and quantitative analysis data were performed using ImageJ, two-sided unpaired t-test. (E) Frequency of fibroblast-like cell in *A. pleuropneumoniae*-infected and control groups analyzed by flow cytometry (control = 3, *A. pleuropneumoniae* = 3). FMO, fluorescence minus one; Isotype, IgG1, κ isotype antibody. Error bars: SEM. *P < 0.05, two-sided Wilcox test. (F) Representative gating strategy for CD45^−^EPCAM^−^CD31^−^SDC2^+^ fibroblast cells in *A. pleuropneumoniae*-infected and control piglet lungs.

**Figure S3.** Violin plots of TLR2 and TLR4 mRNA expression in monocyte and AM from *A. pleuropneumoniae* and control samples. Two-sided Wilcox test.

**Figure S4. Trajectory Analysis of epithelial and fibroblast-like cells in *A. pleuropneumoniae* and control samples.** (A-B) Cells in the tree are colored by the cluster assignment (A) and pseudotime (B). (C) The CytoTRACE score ranges from 0 to 1, where a higher score indicates a lower degree of differentiation, and a lower score indicates a higher degree of differentiation. diff, differentiated.

**Figure S5. The characterization of fibroblast cells in *A. pleuropneumoniae* and control groups.** (A) A UMAP plot highlighting fibroblast-like, proliferating fibroblast and COL3A1 fibroblast clusters. (B) A bubble plot showing the representative GO enrichment terms of the upregulated genes in fibroblast subsets of *A. pleuropneumoniae* compared with control using the human dataset. (C) Cells in the tree are colored by the cluster assignment and pseudotime. (D) Bubble plot showing the expression COL3A1, COL1A1, COL1A2, FN1, VCAN, LUM and DCN in COL3A1 fibroblast from *A. pleuropneumoniae* and control samples. (E) Violin plots of TLR2 mRNA expression in all the fibroblast subsets from *A. pleuropneumoniae* and control samples. Wilcox test.

**Figure 1**


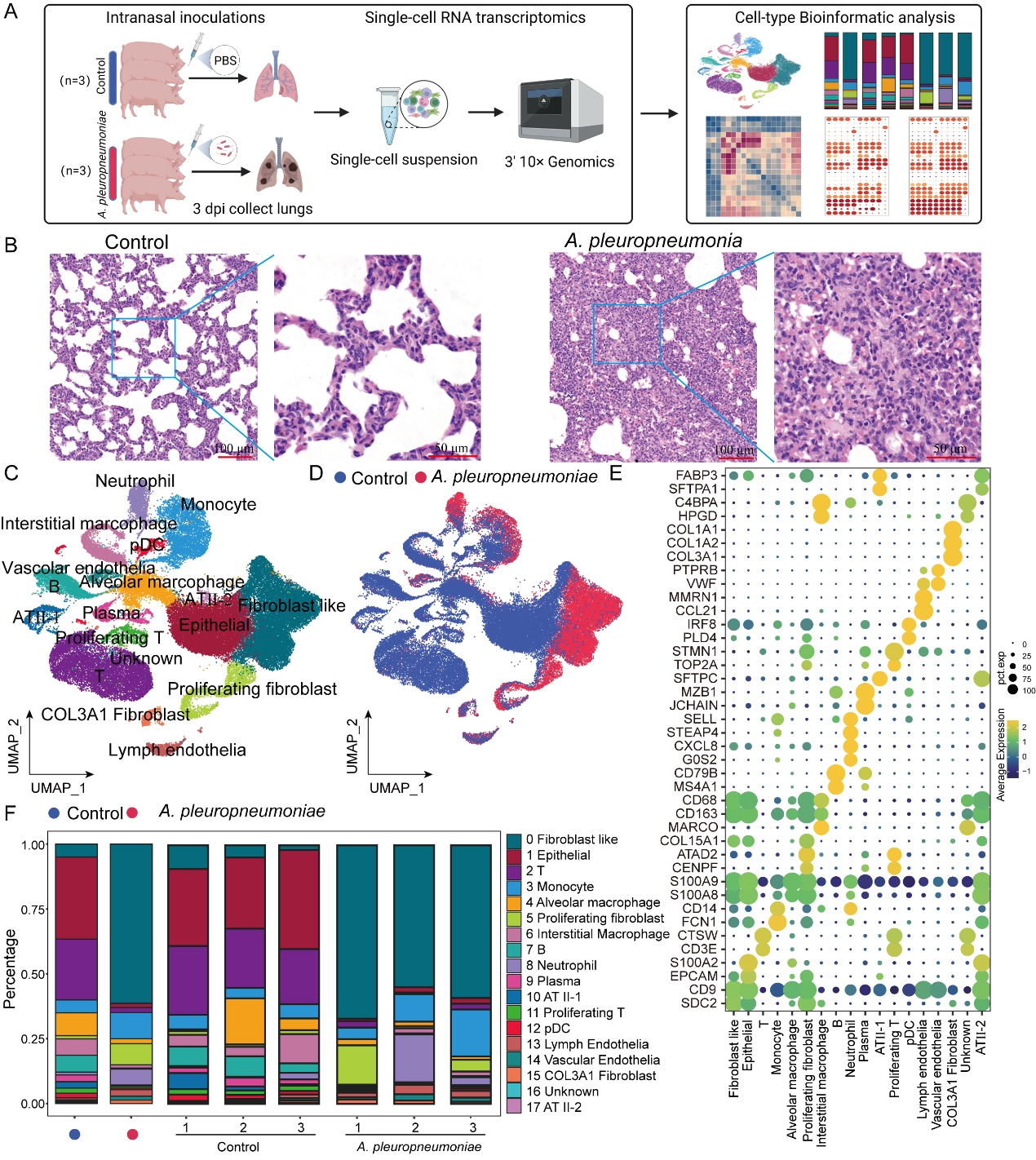


**Figure 2**


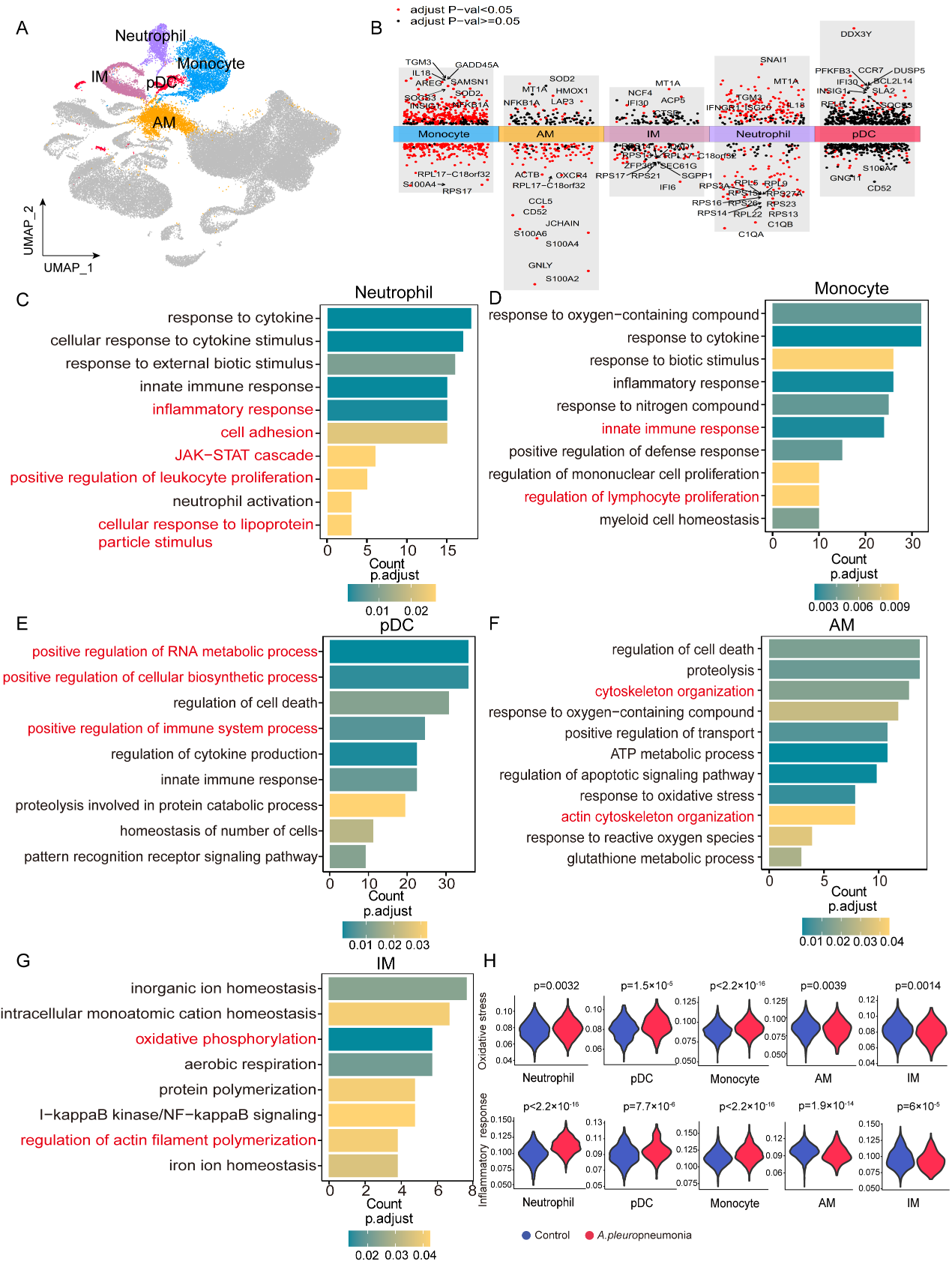


**Figure 3**


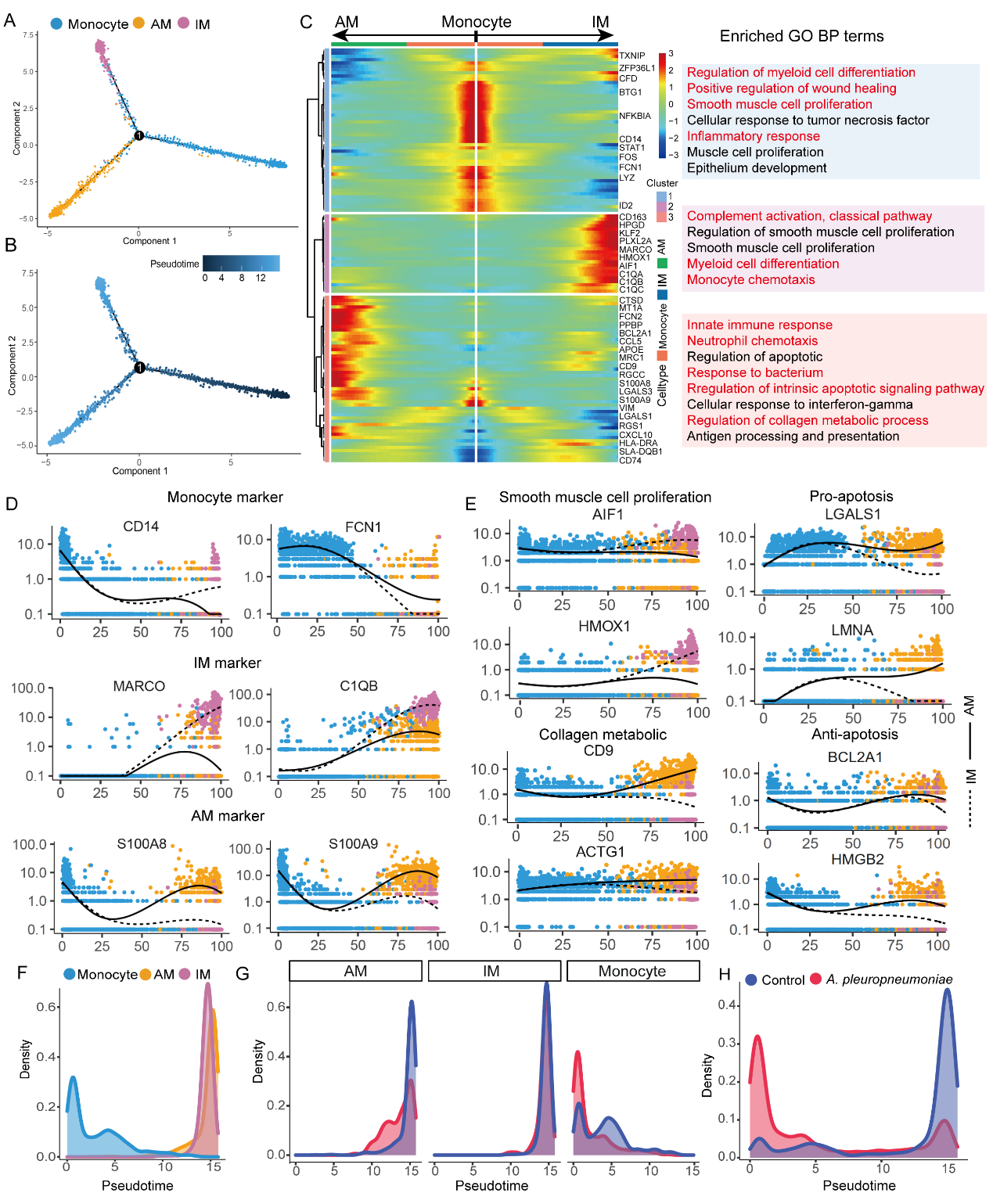


**Figure 4**


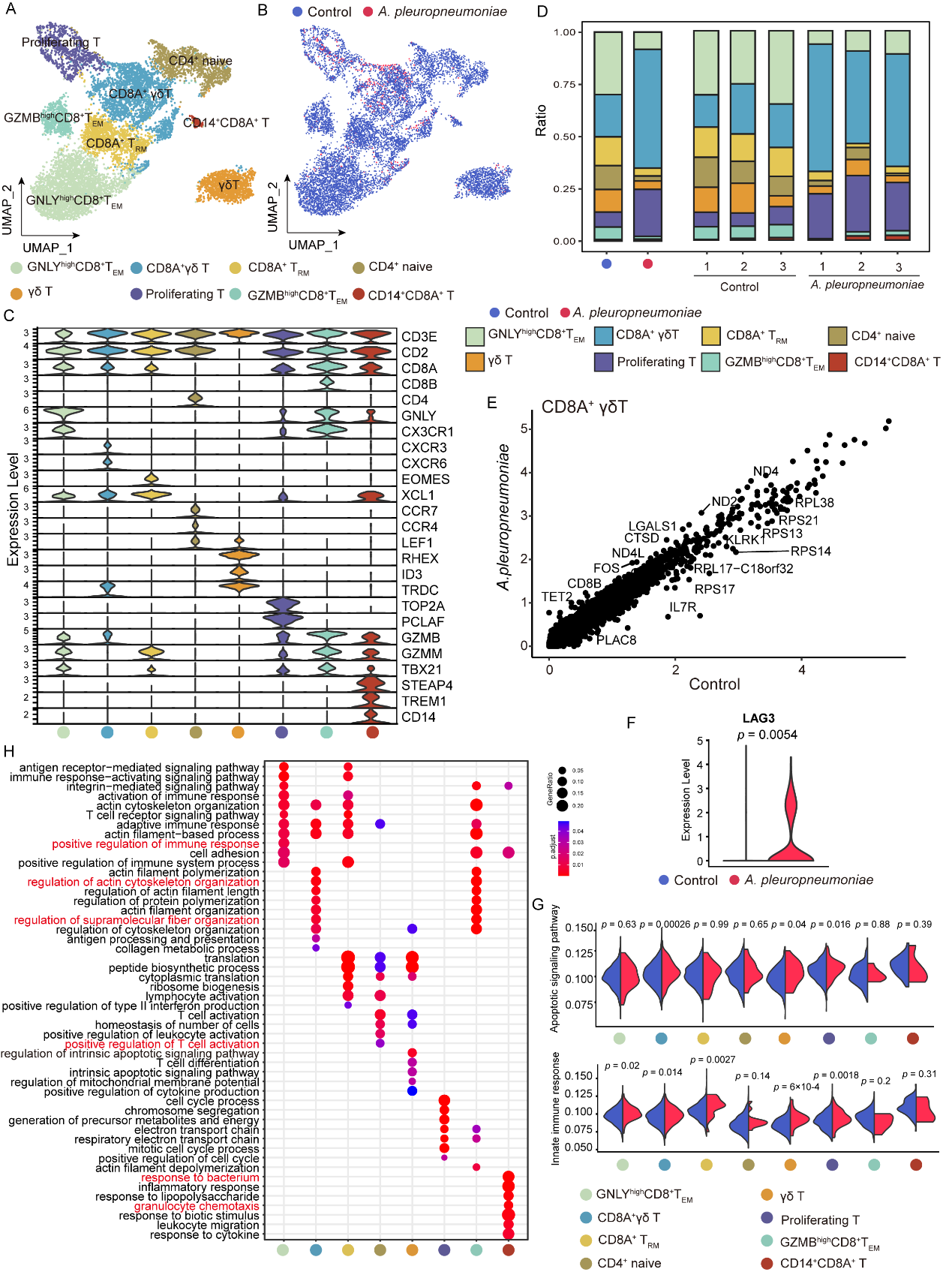


**Figure 5**


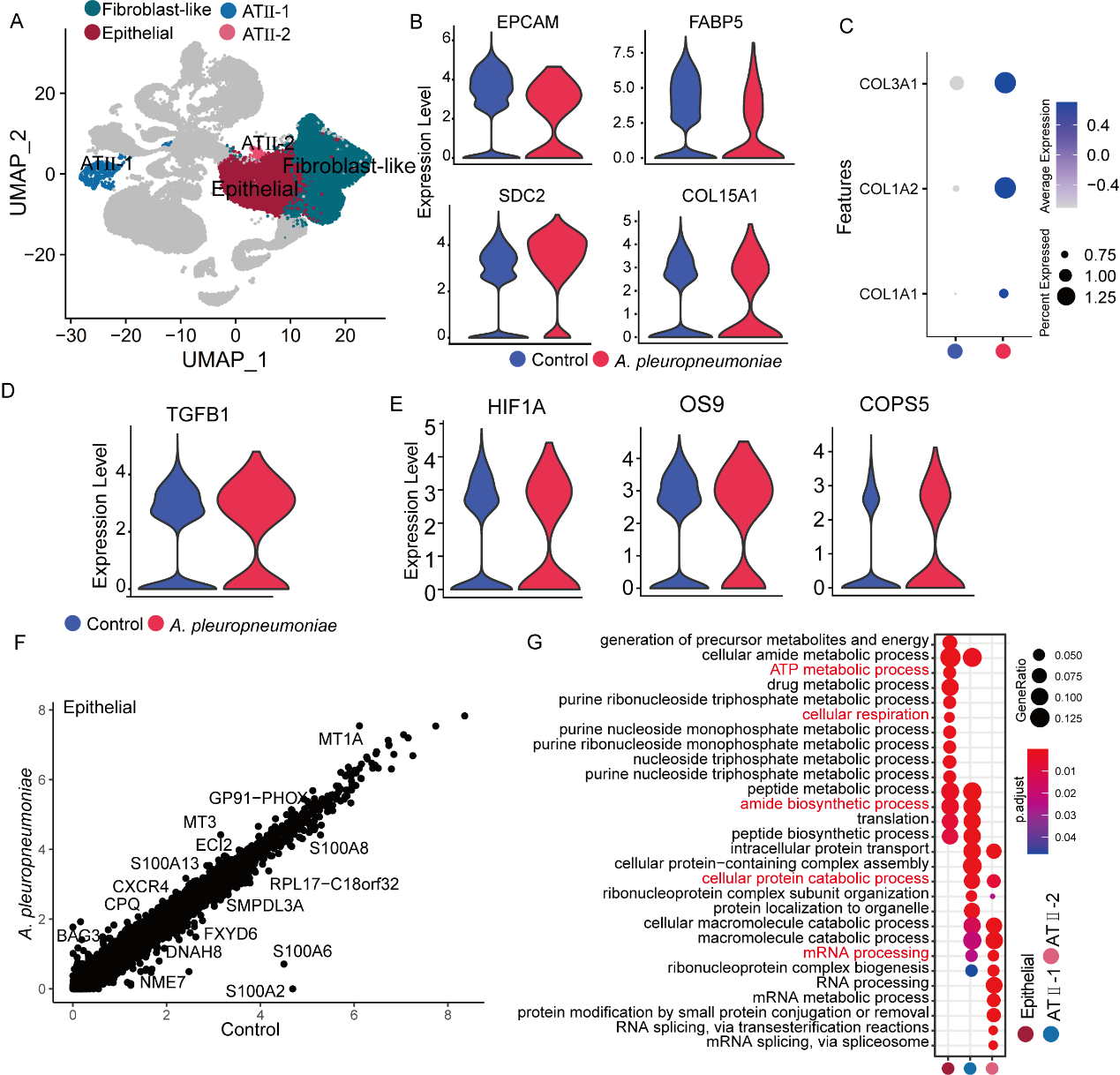


**Figure 6**


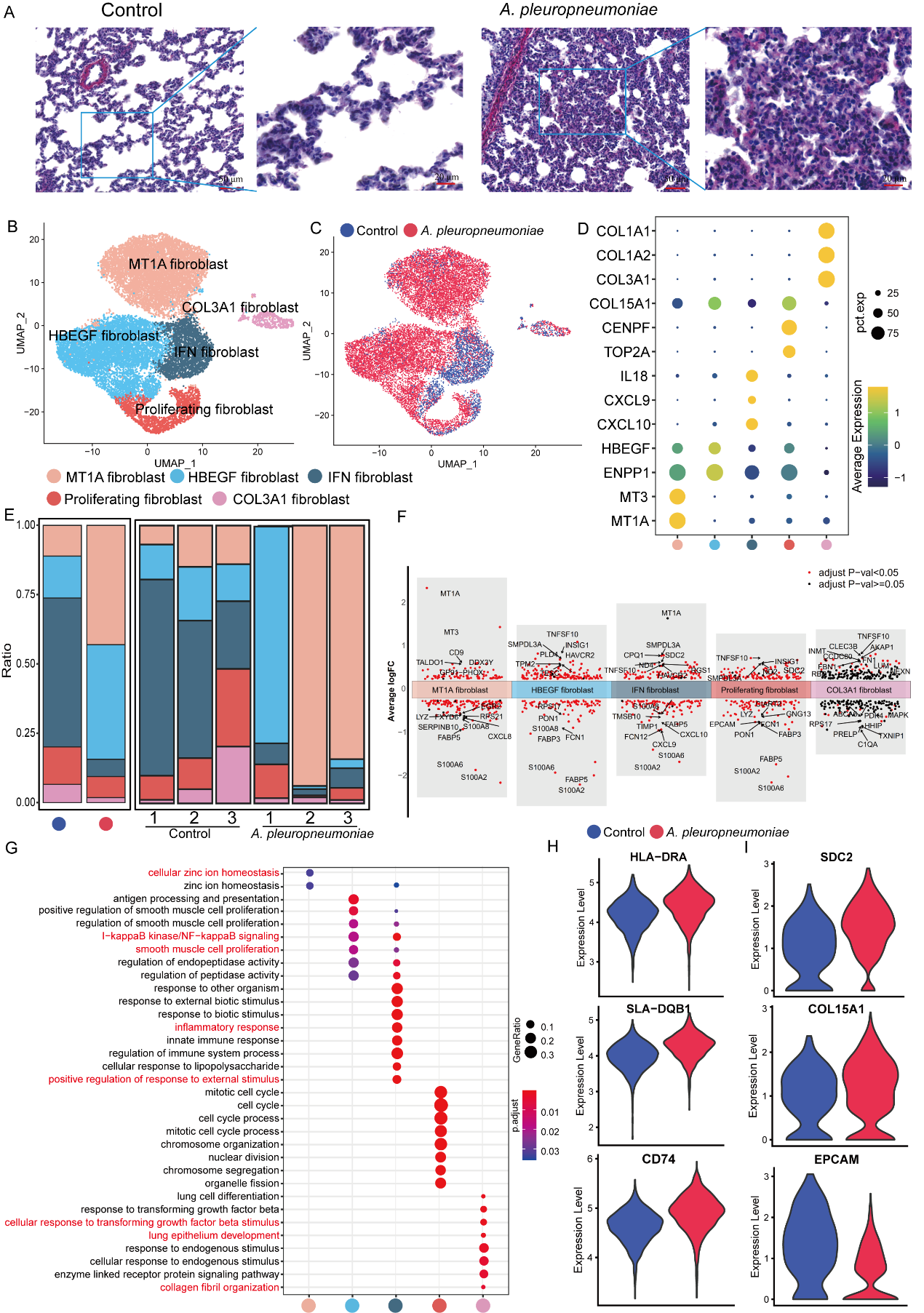


**Figure 7**


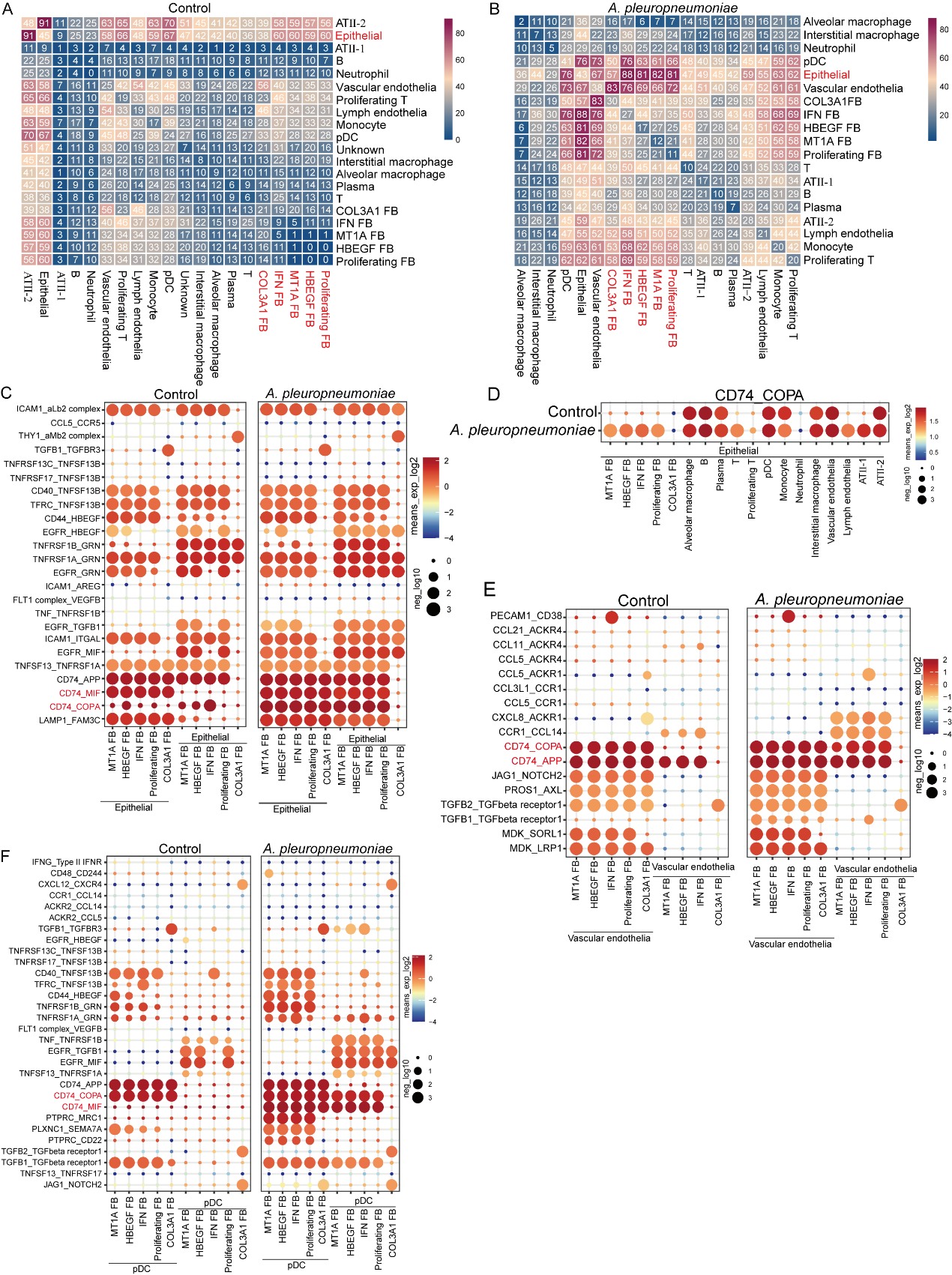


**Figure S1**


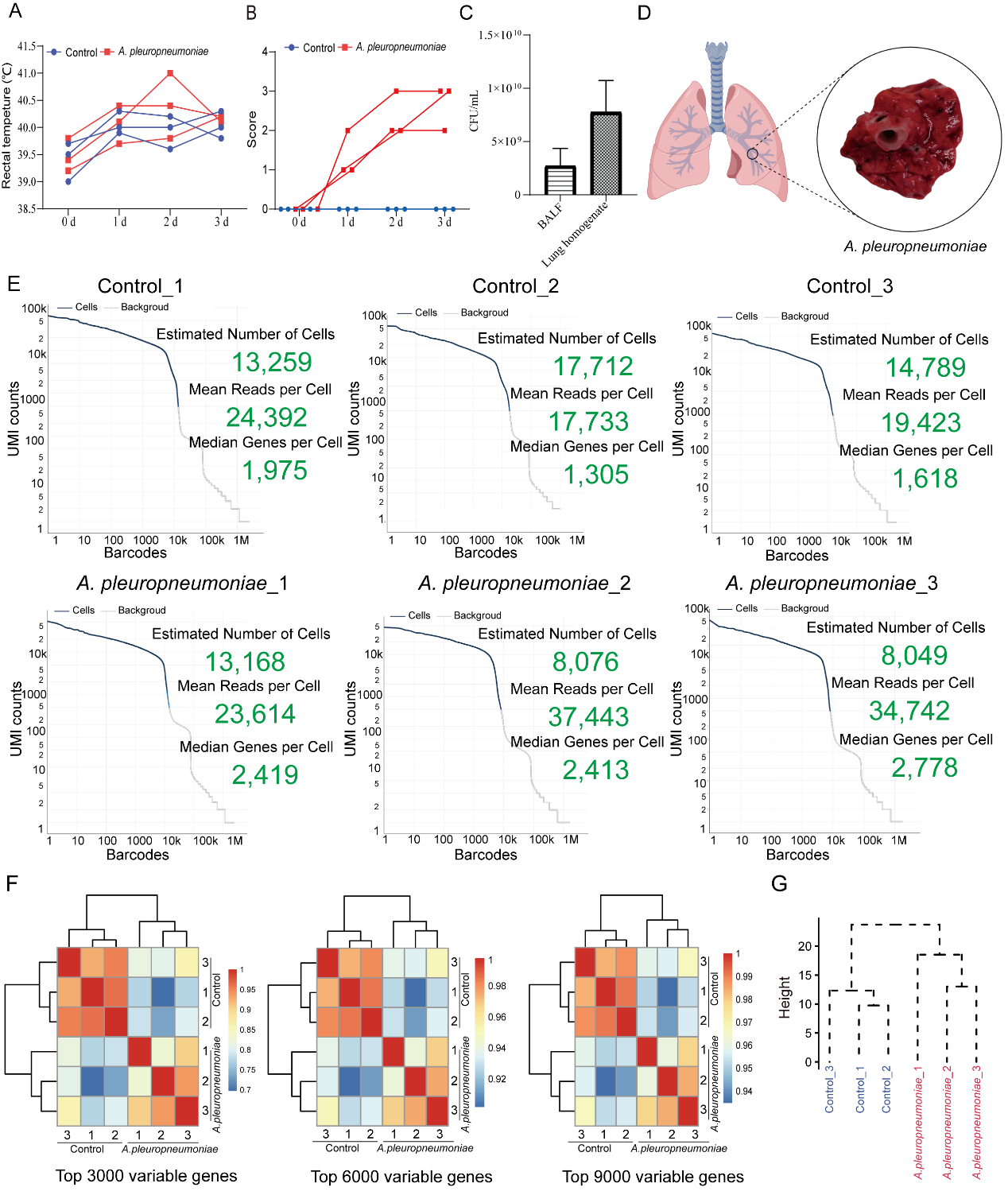


**Figure S2**


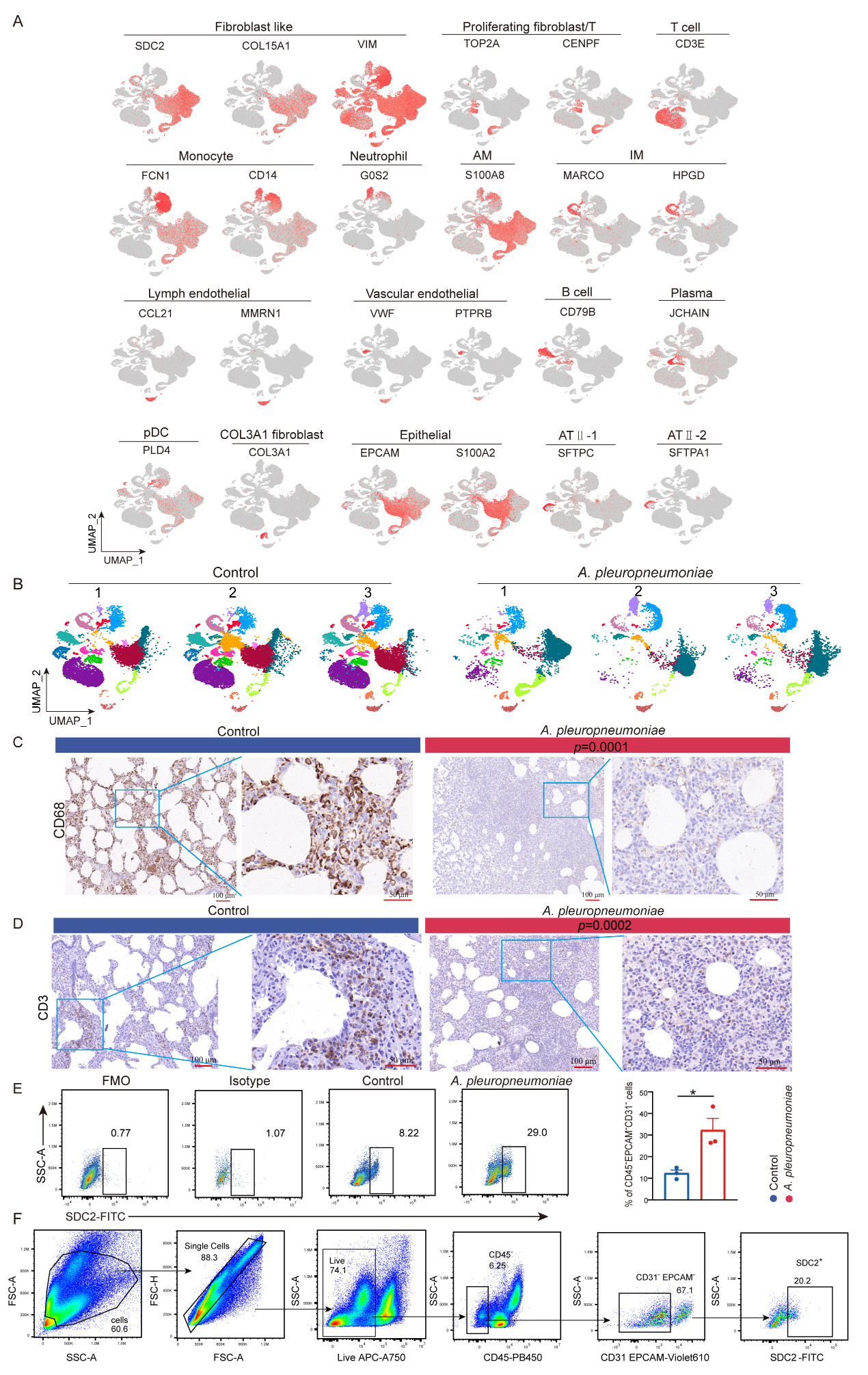


**Figure S3**


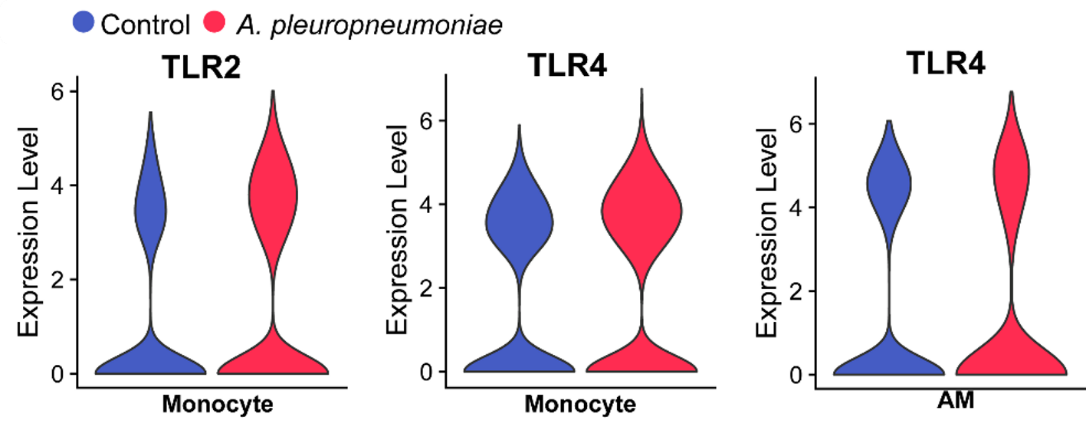


**Figure S4**


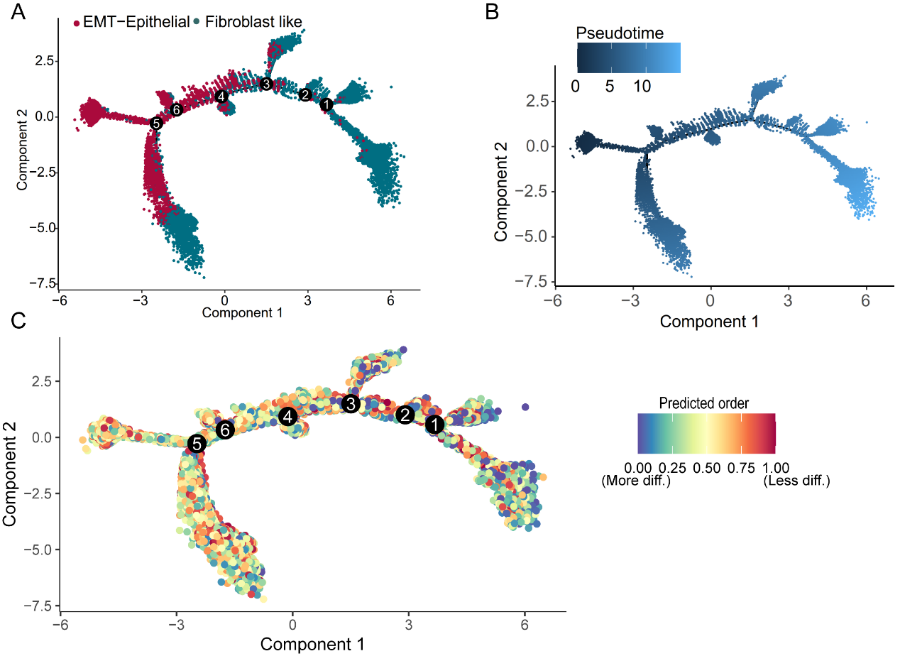


**Figure S5**

**
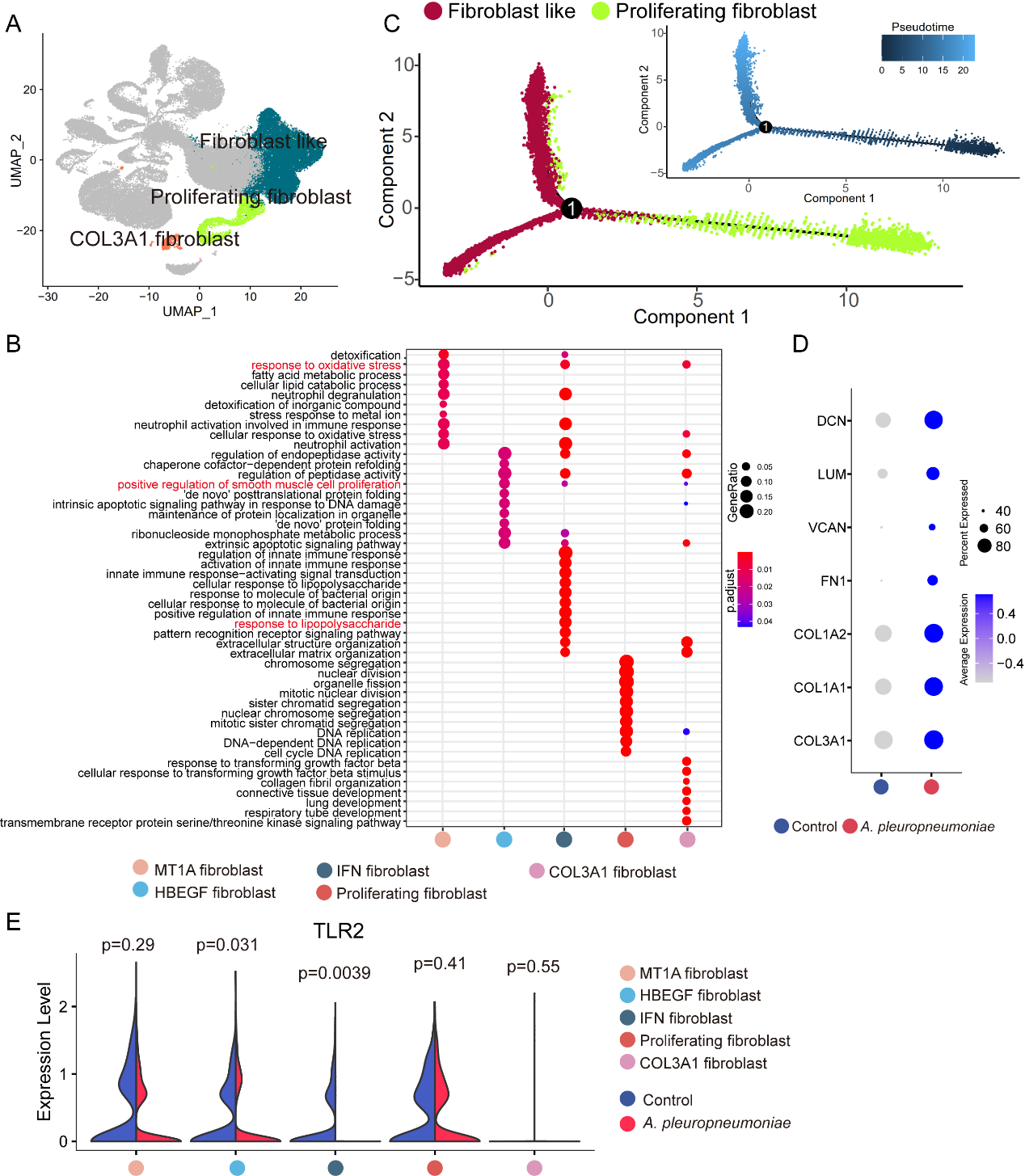
**
